# Mechanisms of nucleic acid degradation and high hydrostatic pressure tolerance of a novel deep-sea wall-less bacterium

**DOI:** 10.1128/mbio.00958-23

**Published:** 2023-08-08

**Authors:** Rikuan Zheng, Chong Wang, Ruining Cai, Yeqi Shan, Chaomin Sun

**Affiliations:** 1 CAS and Shandong Province Key Laboratory of Experimental Marine Biology & Center of Deep Sea Research, Institute of Oceanology, Chinese Academy of Sciences, Qingdao, Shandong, China; 2 Laboratory for Marine Biology and Biotechnology, Qingdao National Laboratory for Marine Science and Technology, Qingdao, Shandong, China; 3 Center of Ocean Mega-Science, Chinese Academy of Sciences, Qingdao, Shandong, China; 4 College of Earth Science, University of Chinese Academy of Sciences, Beijing, China; McMaster University, Hamilton, Ontario, Canada

**Keywords:** deep sea, wall-less bacteria, nucleic acid degradation, high pressure tolerance, chronic bacteriophage

## Abstract

Wall-less bacteria are broadly distributed in diverse habitats. They evolved from a common ancestor within the *Firmicutes* phylum through reductive evolution. Here, we report the cultivation, characterization, and polyphasic taxonomic analysis of the novel free-living wall-less bacterium, *Hujiaoplasma nucleasis* zrk29. We demonstrated that strain zrk29 had a strong ability to degrade DNA and RNA both under laboratory conditions and in the deep sea. We found that nucleic acids induced strain zrk29 to release chronic bacteriophages which supported strain zrk29 and other marine bacteria to metabolize nucleic acids without lysing host cells. We also showed that strain zrk29 tolerated high hydrostatic pressure via two pathways: (i) by transporting cations into its cells to increase intracellular osmotic pressure and (ii) by adjusting the unsaturated fatty acid chain content in its cell membrane phospholipids to increase cell membrane fluidity. This study extends our understanding of free-living wall-less bacteria and provides a useful model to explore the unique adaptation mechanisms of deep-sea microbes.

**IMPORTANCE:**

The unique physiology and survival strategies of the *Tenericutes* bacterium—a typical wall-less bacterium—have fascinated scientists and the public, especially in extreme deep-sea environments where there is high hydrostatic pressure (HHP) and limited availability of nutrients. Here, we have isolated a novel free-living *Tenericutes* strain from deep-sea sediment and have found that it metabolizes nucleic acids with the support of chronic bacteriophages. This *Tenericutes* strain tolerates HHP stress by increasing intracellular osmotic pressure and the unsaturated fatty acid chain content of phospholipids in its cell membrane. Our results provide insights into the unique physiology of deep-sea free-living *Tenericutes* bacteria and highlight the significant role that chronic bacteriophages play in assisting wall-less bacteria to adapt to harsh conditions.

## INTRODUCTION

Members of the orders *Mycoplasmatales*, *Acholeplasmatales*, *Anaeroplasmatales*, *Entomoplasmatales*, *Haloplasmatales*, and *Izemoplasmatales* are wall-less bacteria with small genomes (1 - 3) that are found within a diverse range of environments (4). Currently, the *Izemoplasmatales* order only includes one family: *Izemoplasmataceae. Izemoplasmataceae* is a new family with a free-living lifestyle (5), only one cultured strain, and several unclassified taxa (3). Most studies so far have focused on obligate parasitic wall-less bacteria from the *Mollicutes* class (3), including *Mycoplasmatales*, *Acholeplasmatales*, *Anaeroplasmatales*, and *Entomoplasmatales*, rather than free-living wall-less bacteria. These obligate parasitic bacteria are unique as they have small cell sizes, reduced genome sizes (between 530 and 2,220 kbp) (6, 7), and simplified metabolic pathways (8). Obligate parasitic wall-less bacteria were evolved as a branch of the *Firmicutes* phylum through reductive or degenerative evolution (9). During this process, the obligate parasitic wall-less bacteria lost a considerable portion of their ancestral chromosomes but retained the genes that were essential for life (9). The significant genome compaction that occurred was made possible by the adoption of a parasitic lifestyle. The availability of nutrients from the hosts resulted in the loss of many genes required for assimilative processes during evolution (9). Studying other examples of natural genomic reduction will undoubtedly improve our understanding of the evolutionary forces that drive this process in free-living bacteria (10 - 12). It is still unclear whether other types of genomic reduction exist in free-living wall-less bacteria as most of them remain uncultured and poorly characterized.

Currently, only two free-living wall-less members have been cultured: *Haloplasmatales* and *Izemoplasmatales* representatives (5, 13). A large amount of extracellular DNA is preserved at the surface and subsurface of deep-sea sediments (14), and free-living *Izemoplasmatales* bacteria may be important degraders of this DNA (15, 16). Extracellular DNA contributes substantially to oceanic and sedimentary biogeochemical cycles, acting as an important source of carbon, nitrogen, phosphorus, and energy (17). Free-living *Izemoplasmatales* bacteria may encode multiple extracellular nucleases and nucleotidases involved in the decomposition of extracellular DNA using liberated nucleosides for nutrients and energy. Notably, the only cultured representative of the *Izemoplasmatales* order was isolated from a deep-sea sediment (5, 15). With the harsh deep-sea environment, which includes high hydrostatic pressure (HHP) and a large number of viruses, it is unclear how wall-less bacteria survive, given their small genomes, lack of cell walls, and free-living lifestyle.

Despite the surge of metagenome data available for members of the wall-less bacteria, further research is important to validate their physiology, adaptation strategies, and ecological roles. Here, we successfully cultured a novel deep-sea free-living wall-less bacterium using an innovative approach. Using a combination of genomic, biochemical, transcriptomic, and metabolomic approaches, we revealed the unique mechanisms used by this wall-less bacterium to metabolize nucleic acids and tolerate high hydrostatic pressure. Additionally, we investigated chronic bacteriophages might contribute to nucleic acid utilization, as well as their interactions with their host.

## RESULTS

### Isolation and characterization of the wall-less bacterium, zrk29

A recent study showed that many wall-less bacteria in anoxic marine sediments actively participate in the primary degradation of extracellular DNA polymers (16). We, therefore, tried to isolate more free-living wall-less bacteria that participate in DNA and RNA degradation from different batches of deep-sea samples (Fig. S1A). Based on 16S rRNA gene sequence identity values, we identified one wall-less bacterium, zrk29, belonging to the *Izemoplasmatales* order with the potential to degrade and utilize nucleic acids. Observation by transmission electron microscopy (TEM) showed that the cells of strain zrk29 were coccoid (Fig. S1B and C), approximately 300–500 nm in size, and had no flagellum. Ultrathin TEM sections also showed that the cells of strain zrk29 had no cell wall and contained only a thin membrane in contrast to the Gram-positive bacterium, *Bacillus* sp. zrkA, from the order *Bacillales* (Fig. S1D and E). This confirmed the typical wall-less trait in strain zrk29.

### Genomic characteristics and phylogenetic analysis of strain zrk29

Genomic DNA of strain zrk29 was isolated and sequenced. The genome size of strain zrk29 was 1,808,583 bp with a DNA G + C content of 30.68% (Fig. S2; Table S1). There was only one contig with a total N50 of 1,808,583 bp. The sequencing depth was 491.1×. Annotation of the genome of zrk29 revealed that it consisted of 1,741 predicted genes, which included 45 RNA genes (6 rRNA genes, 36 tRNA genes, and 3 other ncRNAs). The genome relatedness values were calculated using amino acid identity (AAI), average nucleotide identity (ANI), tetranucleotide signatures (Tetra), and *in silico* DNA-DNA similarity (*is*DDH); these calculations were performed against five *Izemoplasmatales* bacterial genomes (zrk29, zrk13, HR1, HR2, and ZiA1). The calculations determined strain zrk29 representing a novel clade within the order *Izemoplasmatales* as its genomic composition is below the established “cut-off” values in delineation of a bacterial species of ≤95% ANIb (use BLASTN+ to align the 1,020-nt fragment of the input sequence), ANIm (use MUMmer to align the input sequence), and AAI, ≤0.99 Tetra, and ≤70% DDH.

**Fig 1:**
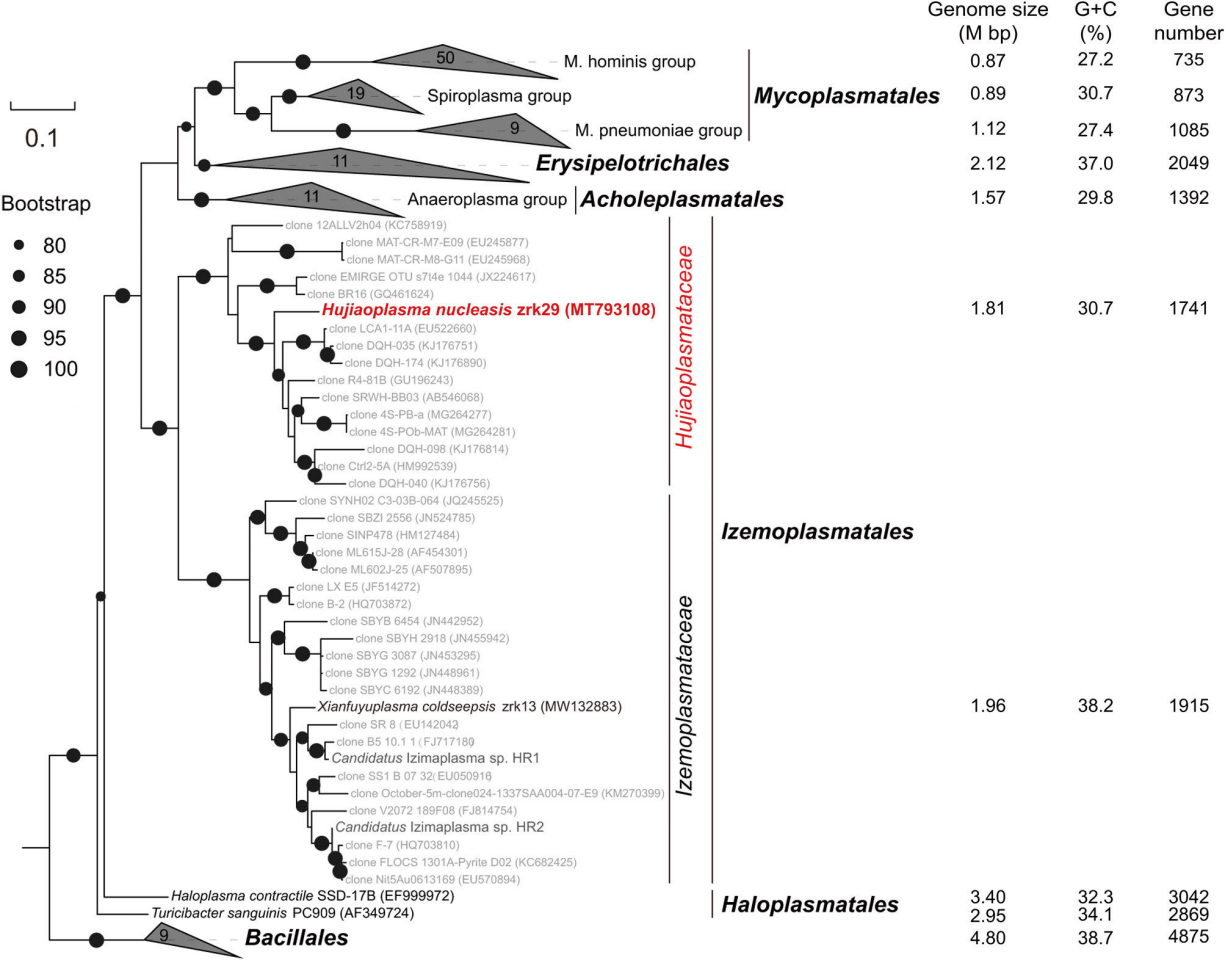
Phylogenetic position and genomic reduction analysis of *Hujiaoplasma nucleasis* zrk29. Maximum likelihood phylogenetic tree of 16S rRNA gene sequences from strain zrk29, other cultured wall-less bacteria, and some *Bacillales* representatives. Bootstrap values (%) >80 are indicated at the base of each node with the black dots (expressed as percentages of 1,000 replications). Genome size, G + C content (%), and gene numbers are shown on the right.

To understand the taxonomic status of strain zrk29, we performed phylogenetic analyses. All the maximum likelihood trees of the 16S rRNA gene sequences (Fig. 1), genomes (Fig. S3), elongation factor Tu (EF-Tu) (Fig. S4), and the beta subunit of RNA polymerase (RpoB) (Fig. S5) supported the placement of the clade of strain zrk29 as a sister group to the *Izemoplasmataceae* family. Notably, the genomes, G + C contents, and gene numbers progressively decreased between *Bacillales* bacteria, free-living, and commensal/parasitic wall-less members—decreasing from 4.80 Mb to 0.87 Mb, 38.7% to 27.2%, and 4,875 to 735, respectively (Fig. 1; Data set S1). These results suggested that the clades of strain zrk29 and *Izemoplasmataceae* could represent key intermediates in the reductive evolution from *Bacillales* to obligate parasitic wall-less bacteria. Moreover, a sequence similarity calculation using the NCBI server indicated that the closest relative of strain zrk29 was *Haloplasma contractile* SSD-17B (85.68%) and *Xianfuyuplasma coldseepsis* zrk13 (86.03%); these values were all lower than the sequence identity threshold of 86.5% for distinct families (18). Therefore, we proposed the name *Hujiaoplasmataceae* for the novel family. Strain zrk29 was the first free-living representative of this clade. Based on these criteria, we proposed the names, *Hujiaoplasmataceae* fam. Nov. and *Hujiaoplasma nucleasis* gen. nov., sp. nov.

### *H. nucleasis* zrk29 possesses a strong ability to metabolize both DNA and RNA

**Fig 2:**
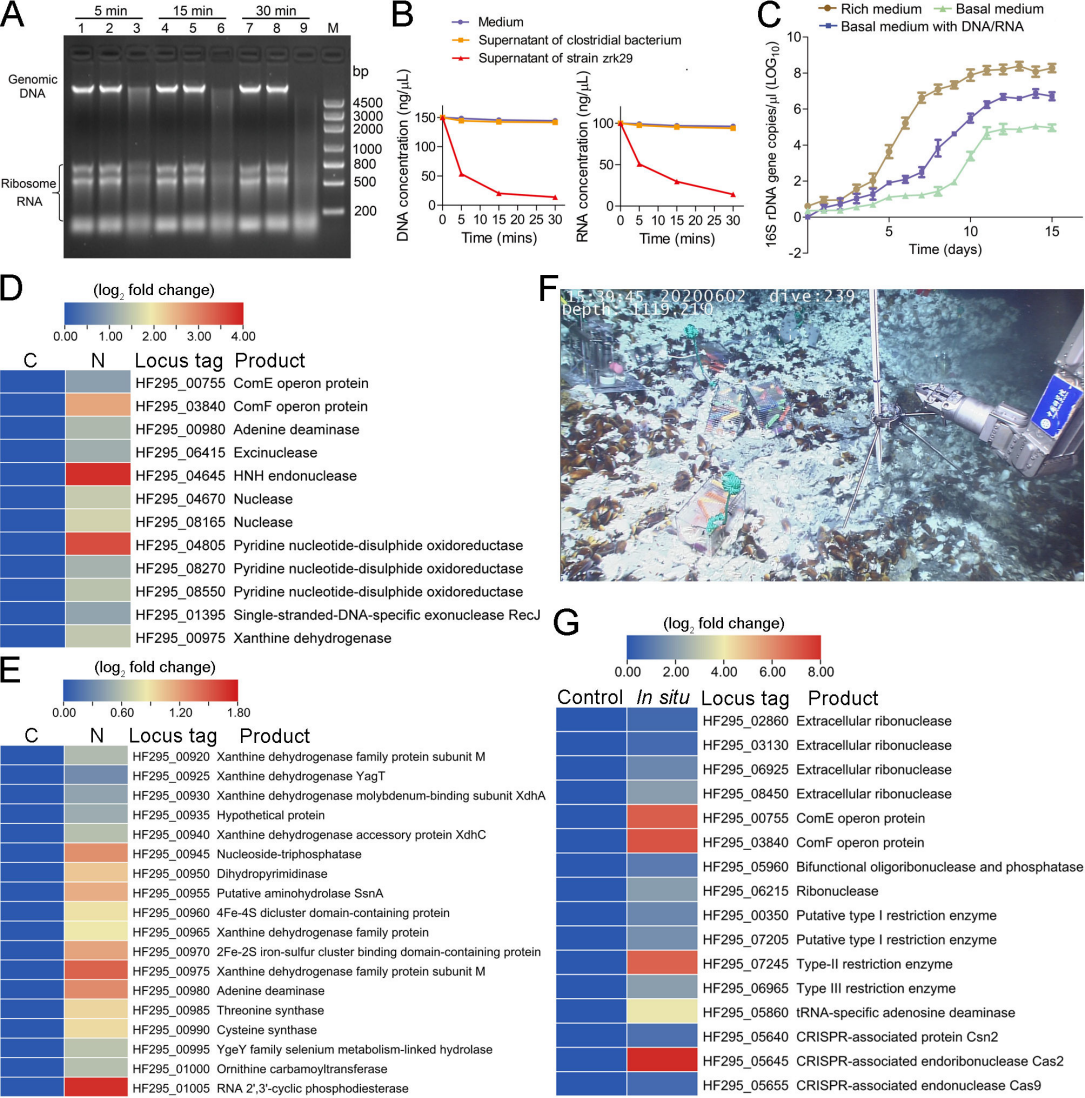
*H*. *nucleasis* zrk29 has a strong ability to degrade and utilize DNA and RNA. (**A**) Lane 1, lane 4, and lane 7 indicate 2.5 µg of *E. coli* genomic DNA/RNA treated with medium alone for 5 minutes, 15 minutes, and 30 minutes at 37°C, respectively. Lane 2, lane 5, and lane 8 indicate 2.5 µg of *E. coli* genomic DNA/RNA treated with the culture supernatant of a *Clostridia* bacterium for 5 minutes, 15 minutes, and 30 minutes at 37°C, respectively. Lane 3, lane 6, and lane 9 indicate 2.5 µg of *E. coli* genomic DNA/RNA treated with the culture supernatant of strain zrk29 for 5 minutes, 15 minutes, and 30 minutes at 37°C, respectively. M, DL5000 molecular weight DNA marker. (**B**) Quantification of DNA and RNA degradation by strain zrk29. (**C**) Growth assays of strain zrk29 cultivated in either rich medium, basal medium alone, or basal medium supplemented with 100 µg/mL *E. coli* genomic DNA and 100 µg/mL RNA. (**D**) Laboratorial transcriptomics-based heat map showing all upregulated genes encoding enzymes with nucleic acid degradation abilities. “C” indicates strain zrk29 cultivated in basal medium; “N” indicates strain zrk29 cultivated in basal medium supplemented with 100 µg/mL *E. coli* genomic DNA and 100 µg/mL RNA. (**E**) Laboratorial transcriptomics-based heat map showing an upregulated gene cluster encoding nucleotide and amino acid metabolism-associated proteins and Fe-S protein. (**F**) Close views of the *in situ* experimental apparatus in the deep-sea cold seep where many mussels and shrimps were distributed. (**G**) *In situ* transcriptomics-based heat map showing all upregulated genes encoding enzymes with nucleic acid degradation abilities. “Control” indicates strain zrk29 cultivated in a deep-sea cold seep without exchange with outside; “*In situ*” indicates strain zrk29 cultivated in a deep-sea cold seep with exchange with outside. Three replicates were performed.

As previously reported, members of the order *Izemoplasmatales* are specialized DNA degraders (3, 5, 16). Combined with our current isolation strategy, we speculated that strain zrk29 might also be a specialized nucleic acid degrader. To verify this hypothesis, we tested for DNA and RNA degradation and utilization in strain zrk29. Strikingly, in the culture supernatant of strain zrk29, we found that *Escherichia coli* DNA and RNA had been degraded within 5 minutes (Fig. 2A, lane 3), with almost complete degradation after 30 minutes at 37°C (Fig. 2A, lane 9). We found that rRNAs were only partially degraded, indicating that strain zrk29 might inefficiently degrades the structured RNAs. Quantitatively, strain zrk29 had decreased the concentration of DNA from 150 ng/µL to 13.6 ng/µL and the concentration of RNA from 100 ng/µL to 14.1 ng/µL (Fig. 2B). The pattern of DNA and RNA degradation by strain zrk29 at 4°C was similar to that at 37°C (Fig. S6), but the rate of degradation was slower (taking approximately 6–10 times longer). When the basal medium was supplemented with DNA and RNA, this significantly promoted the growth rate and biomass of strain zrk29 compared to cultures grown in basal medium alone (Fig. 2C). This strongly suggests that strain zrk29 uses DNA and RNA for nutrients to support its growth. The growth rate of strain zrk29 was also promoted in the rich medium containing yeast extract (1.0  g/L) and peptone (1.0  g/L) when compared to that cultured in the basal medium alone or basal medium supplemented with DNA and RNA, indicating strain zrk29 is a heterotrophic bacterium (Fig. 2C). Analysis of the genome of strain zrk29 showed the presence of a putative nucleic acid-degradation locus (Fig. S7A). This locus contained genes encoding extracellular ribonuclease, bifunctional oligoribonuclease/PAP phosphatase NrnA, a nucleoid-associated protein, ABC transporters, and other proteins associated with nucleic acid degradation. Subsequently, we used qRT-PCR to investigate the gene expression within this nucleic acid-degradation locus for strains of zrk29 cultured in basal medium with DNA and RNA compared to strains cultivated in basal medium alone. We found that many genes within this locus were significantly upregulated (Fig. S7B). In particular, genes encoding extracellular ribonuclease, recombination protein RecR, and a hypothetical protein were upregulated by approximately 27-fold (Fig. S7B), indicating their key roles in nucleic acid degradation and utilization.

Furthermore, we performed comparative transcriptomic analyses for strains of zrk29 cultured in either basal medium supplemented with DNA and RNA or basal medium alone. As expected, we observed a notable upregulation in the expression of key genes encoding enzymes closely associated with nucleotide metabolism, including nuclease, HNH endonuclease, adenine deaminase, xanthine dehydrogenase, and ComE/ComF (used for the binding and uptake of nucleic acids) (Fig. 2D). A gene cluster containing genes encoding Fe-S cluster proteins and various proteins associated with nucleotide and amino acid metabolism (from HF295_00920 to HF295_01005) were also significantly upregulated (Fig. 2E). The Fe-S cluster proteins are metal cofactors which are required for respiration, DNA repair, nucleotide metabolism, and other biological pathways (19). Notably, most factors associated with nucleotide metabolism were xanthine dehydrogenase-related proteins (Fig. 2E) belonging to the molybdenum-containing group of hydroxylases, which are involved in purine metabolism. One xanthine dehydrogenase was present between the two Fe-S proteins; this might form an operon with the adjacent iron-sulfur binding proteins to participate in nucleic acid degradation. The adjacent Fe-S proteins might also be electron-transferring components of these xanthine dehydrogenases (20). In addition, we also found a significant upregulation in the expression of genes encoding nucleoside-triphosphatase, dihydropyrimidinase, and adenine deaminase (which are associated with nucleotide metabolism). Thus, we speculate that nucleic acid degradation might be facilitated by Fe-S-associated proteins to create energy and promote growth in strain zrk29.

**Fig 3:**
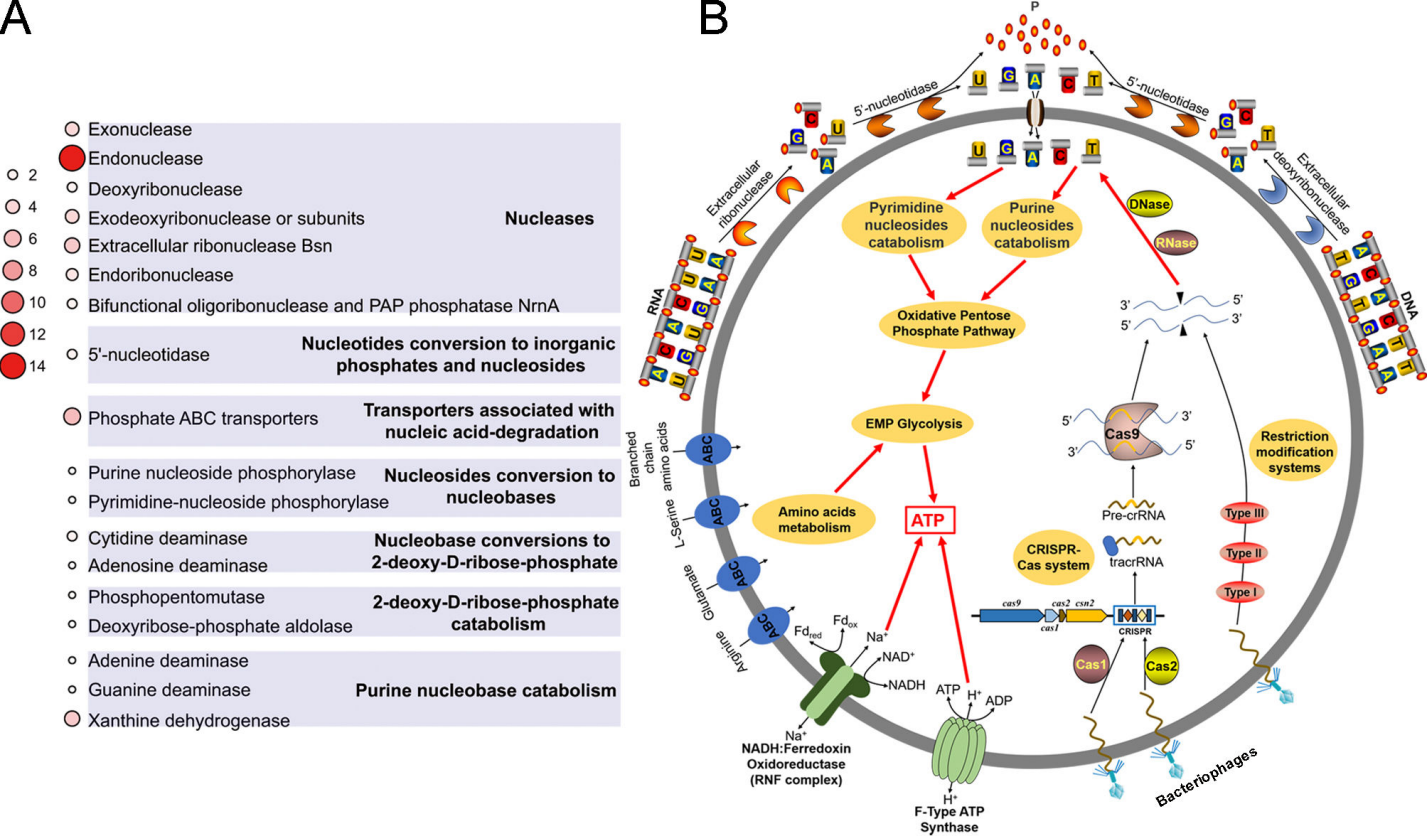
Nucleic acid metabolic pathway of *H. nucleasis* zrk29. (**A**) Statistical analysis of key genes encoding enzymes involved in nucleic acid degradation, transport, and catabolism of nucleic acid subcomponents in the genome of strain zrk29. The number of genes encoding enzymes is indicated by the size and shade of red circles. (**B**) A proposed model of central metabolism associated with nucleic acid degradation and utilization by strain zrk29. The contribution of defense systems, including the CRISPR and RM systems, are also included.

The above results suggested that strain zrk29 had a strong ability to degrade and utilize DNA and RNA under laboratory conditions; however, its nucleic acid degradation abilities in the deep-sea environment were still unclear. We, therefore, cultivated strain zrk29 in a deep-sea cold seep for 10 days in June 2020 using culture apparatus (Fig. 2F). We then investigated its *in situ* metabolism via a transcriptomic method. The *in situ* transcriptomic results showed up to 921 differentially expressed genes compared to the control group (Fig. S8A). Many GO categories associated with nucleic acid degradation were significantly enriched among the upregulated genes, including nucleic acid binding, nuclease activity, purine ribonucleotide metabolism, and nucleotide metabolic processes, among others (Fig. S8B). The expression of all genes involved in Embden-Meyerhof-Parnas (EMP) glycolysis was significantly downregulated (Fig. S8C and D), which could be due to a lack of organic nutrients, like saccharides, in the deep-sea environment. Among the upregulated nucleic acid degradation genes, *comE* and *comF*—which are responsible for the binding and uptake of DNA—showed up to a 120-fold increase in expression (Fig. 2G), similar to our observations under laboratory conditions (Fig. 2D). Moreover, many genes encoding extracellular ribonuclease were also upregulated (Fig. 2G), indicating that strain zrk29 might degrade extracellular DNA and RNA and then transport the nucleotides into its cells for further metabolism (Fig. 3). Surprisingly, we also found upregulated expression for genes encoding restriction enzymes (Type I, Type II, and Type III) associated with restriction and modification (RM) systems, and CRISPR-associated endonuclease Cas9, endoribonuclease Cas2, and Csn2. This indicates that RM and CRISPR-Cas systems of strain zrk29 are functional in the deep sea. The CRISPR-Cas system present in strain zrk29 was identified as Type II-A (Fig. S9A); it is composed of four *cas* genes (*cas9*, *cas1*, *cas2*, and *csn2*), and a CRISPR locus with spacers of 30 bp and direct repeats of 36 bp (Fig. S9B). However, after searching phage, plasmid, and virus databases using CRISPRTarget, none of the 40 spacers yielded any significant results. Taken together, we speculate that strain zrk29 has evolved to a sophisticated defense system, including CRISPR and RM systems, to avoid the harmful effects of phages (Fig. 3).

### Nucleic acids induce the release of a chronic bacteriophage from strain zrk29

**Fig 4:**
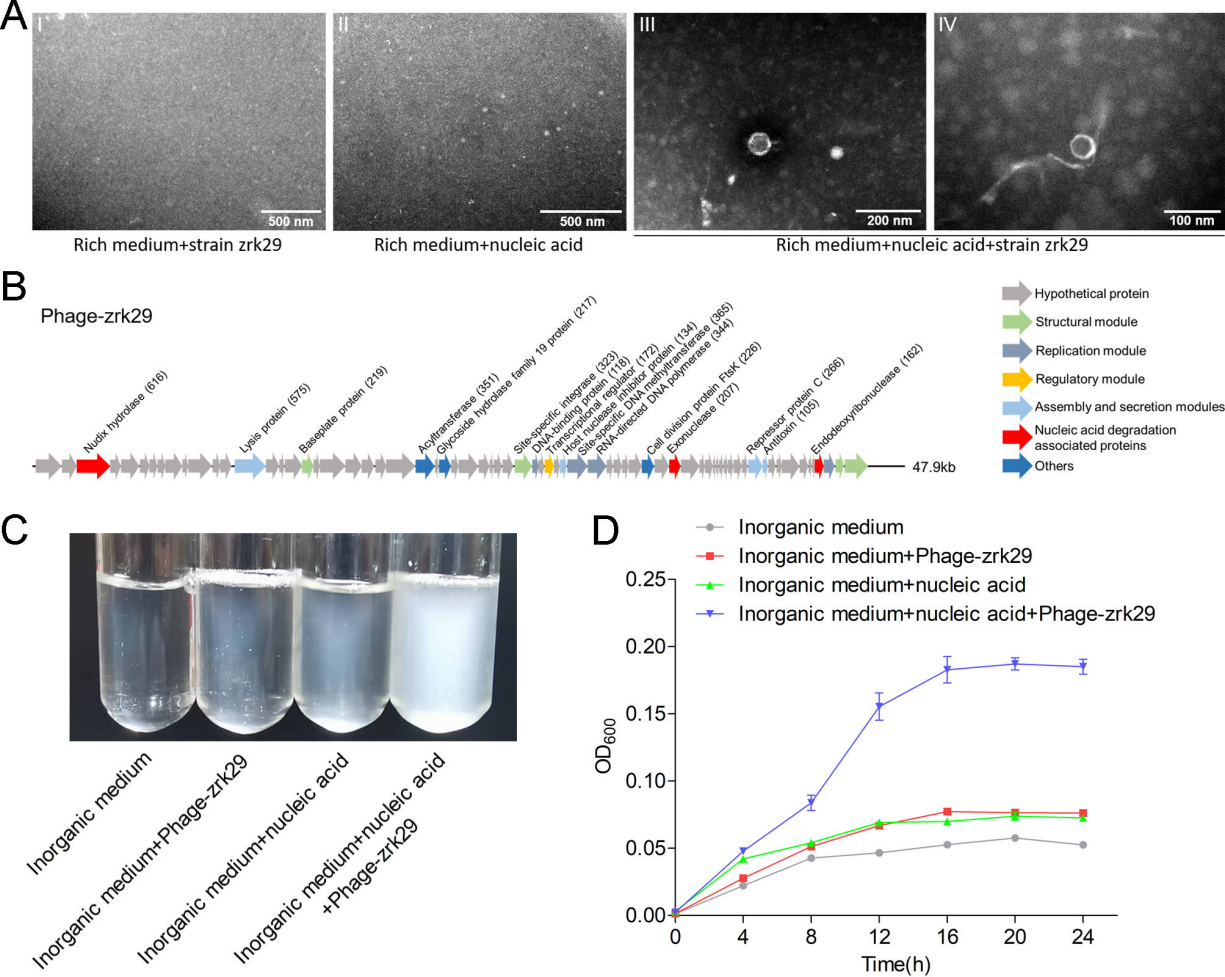
The chronic bacteriophage Phage-zrk29 isolated from *H. nucleasis* zrk29 hydrolyzes nucleic acids. (**A**) TEM observation of phages extracted from the culture supernatant of zrk29 strains cultured in the rich medium supplemented with 100 µg/mL *E. coli* genomic DNA and 100 µg/mL RNA and zrk29 strains cultivated in rich medium alone. Panels I and II show that no phage-like particles were observed in the culture supernatant of zrk29 strains cultured in the rich medium or the rich medium supplemented with nucleic acids but not inoculated with strain zrk29, respectively. Panels III and IV show that hexagonal phages were observed in the culture supernatant of the zrk29 strains cultured in the rich medium supplemented with nucleic acids. (**B**) A diagram showing the genomic composition of Phage-zrk29. Arrows represent different ORFs and the direction of transcription. The main putative gene products of Phage-zrk29 are shown. The numbers of amino acids are indicated in brackets. The hypothetical proteins are indicated by gray arrows, the structural modules are indicated by green arrows, the replication module is indicated by blue-gray arrows, the regulatory module is indicated by golden arrows, and the assembly and secretion modules are indicated by blue arrows. Nucleic acids degradation-associated proteins are indicated by red arrows, and other proteins are indicated by dark blue arrows. The phage genome size is shown behind the gene cluster. The growth status (**C**) and growth curve (**D**) of *Pseudomonas stutzeri* 273 cultivated in inorganic medium alone, inorganic medium supplemented with 20 µL/mL Phage-zrk29, inorganic medium supplemented with nucleic acids, and inorganic medium supplemented with nucleic acids and 20 µL/mL Phage-zrk29.

Some non-specific nucleases encoded by bacteriophages have recently been proposed to possess both DNA and RNA degradation abilities (21, 22). As strain zrk29 is a wall-less bacterium that is small in size, we speculated that its ability to metabolize nucleic acids might be facilitated by phages. TEM observation showed that hexagonal phages-like structures (30–40 nm) were indeed present in the culture supernatant of the zrk29 strains that were cultured in the rich medium supplemented with nucleic acids (Fig. 4A, panels III-IV). In contrast, we did not observe any phages-like structures in the two control groups, which included the supernatant of the rich medium supplemented with nucleic acid but not inoculated with strain zrk29 (Fig. 4A, panel II) and the culture supernatant of zrk29 strains that were cultured in the rich medium alone (Fig. 4A, panel I). These results suggest that it was the presence of the nucleic acids that induced the release of bacteriophages from strain zrk29. Moreover, the release of bacteriophages from strain zrk29 did not negatively affect its growth, consistent with the key feature of chronic phages (23, 24).

We subsequently sequenced the genome of the chronic bacteriophage (Phage-zrk29) that was induced by the presence of nucleic acids from strain zrk29. We compared the phage genome (Phage-zrk29, 47.9 kb) with the host genome (strain zrk29) using Galaxy version 2.6.0 (https://galaxy.pasteur.fr/) (25) with the NCBI BLASTN method. We found that the Phage-zrk29 genome was completely outside of the host chromosome, indicating that this chronic phage was extrachromosomal, consistent with previous reports (26). In addition to genes encoding phage-associated proteins, the genome of Phage-zrk29 also had many auxiliary metabolic genes (AMGs) encoding exonuclease, endodeoxyribonuclease, and nudix hydrolase (Fig. 4B); these proteins might facilitate nucleic acid metabolism in the host cells. To test our hypothesis, we investigated whether the presence of Phage-zrk29 could promote the growth of a marine bacterium, *Pseudomonas stutzeri* 273 (27). We found that Phage-zrk29 significantly promoted the growth of strain 273 by facilitating the utilization of nucleic acids (Fig. 4C). Specifically, supplementing inorganic medium with both Phage-zrk29 and nucleic acids resulted in an approximately threefold increase in growth compared to strains that were cultivated in inorganic medium alone, inorganic medium supplemented with Phage-zrk29 but without nucleic acids, or inorganic medium supplemented with nucleic acids but without Phage-zrk29 (Fig. 4D). These results together suggested that the chronic phage, Phage-zrk29, facilitated the host’s ability to metabolize nucleic acids.

### *H. nucleasis* zrk29 tolerates HHP by adjusting cellular osmotic pressure and cell membrane components

**Fig 5:**
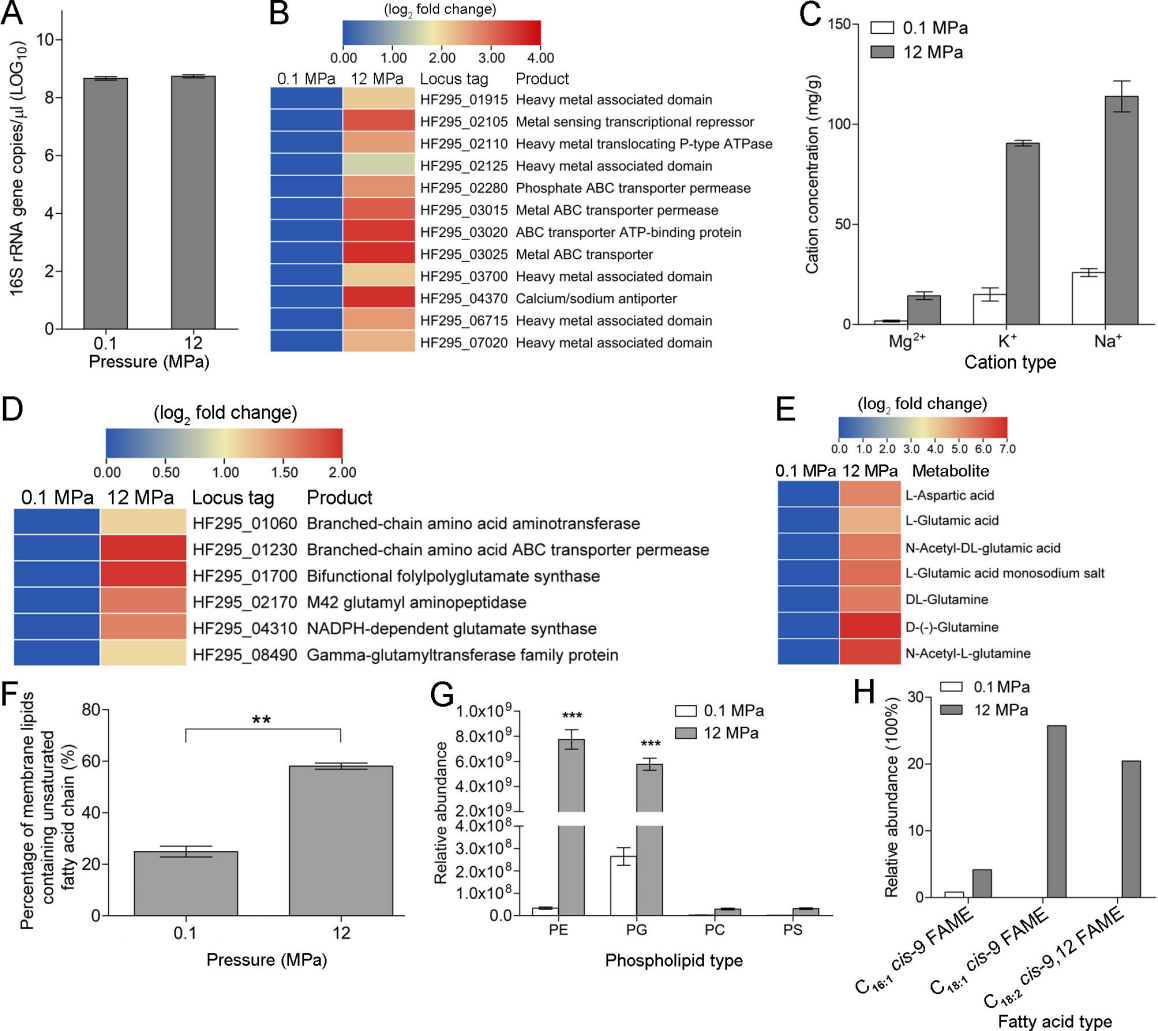
*H*. *nucleasis* zrk29 tolerates high hydrostatic pressure by adjusting cellular osmotic pressure and the proportion of lipids that contain unsaturated fatty acid chains. (**A**) Growth assay of strain zrk29 cultivated either under 0.1 MPa or 12 MPa. (**B**) Transcriptomics-based heat map showing upregulated genes associated with cation transporters. (**C**) Detection of intracellular cation concentrations (Mg^2+^/ K^+^/Na^+^) in strain zrk29 cultivated under 0.1 MPa or 12 MPa, respectively. (**D**) Transcriptomics-based heat map showing upregulated genes associated with amino acid transporters. (**E**) Metabolomics-based heat map showing upregulated metabolites associated with negatively charged amino acids. (**F**) Changes in the proportion of lipids containing unsaturated fatty acid chains in strain zrk29 incubated under 0.1 MPa or 12 MPa. (**G**) Changes in the relative abundance of different phospholipids containing unsaturated fatty acid chains of strain zrk29 incubated either under 0.1 MPa or 12 MPa. PE, phosphatidyl ethanolamines; PG, phosphatidyl glycerol; PC, phosphatidyl choline; PS, phosphatidyl serine. (**H**) Changes in the cellular fatty acids content of strain zrk29 incubated under 0.1 MPa and 12 MPa.

As we isolated the wall-less bacterium, strain zrk29, from the deep sea (HHP: ~12 MPa), we next explored the mechanisms that strain zrk29 uses to tolerate HHP. To investigate this, we used a high-pressure device to culture strain zrk29, simulating the same HHP (12 Mpa) of the deep sea. The result showed that strain zrk29 growth rates under high pressure and atmospheric pressure (0.1 Mpa) were similar (Fig. 5A). To further explore the mechanisms used by strain zrk29 to tolerate HHP, we performed transcriptomic and metabolomic analyses for strains of zrk29 cultured under different pressures (12 MPa and 0.1 MPa). The transcriptomic results revealed that the expression of genes encoding metal ABC transporters, heavy metal translocating P-type ATPase, and a calcium/sodium antiporter was significantly upregulated (Fig. 5B). This suggests that strain zrk29 might transport ions into its cell to increase osmotic pressure and therefore counteract the HHP. Indeed, we found that the intracellular concentrations of magnesium ions (Mg^2+^), potassium ions (K^+^), and sodium ions (Na^+^) were much higher (by approximately four to eight times) in strains of zrk29 cultivated under 12 MPa than in strains cultivated under 0.1 MPa (Fig. 5C). In addition, we observed a markedly upregulated expression of genes encoding branched-chain amino acid ABC transporter permeases, NADPH-dependent glutamate synthases, glutamyl aminopeptidases, and gamma-glutamyltransferase family proteins (Fig. 5D). However, the yields of some metabolites associated with glutamate synthesis were significantly increased. These might be involved in the synthesis and transport of negatively charged amino acids, such as glutamic acid and glutamine. As expected, our metabolomics assays showed that amounts of aspartic acid, glutamic acid, and glutamine were significantly higher in the cells from strains of zrk29 cultured under 12 Mpa compared with strains cultured under 0.1 Mpa (Fig. 5E). This suggests that these amino acids may be balancing the strong positive charges of the cations, thereby preventing cellular damage. Strain zrk29 might, therefore, respond to high osmotic stress by increasing its intracellular concentration of cations, while simultaneously importing from the outside or synthesizing compatible solutes, such as glutamic acid, glutamine, and aspartic acid.

**Fig 6:**
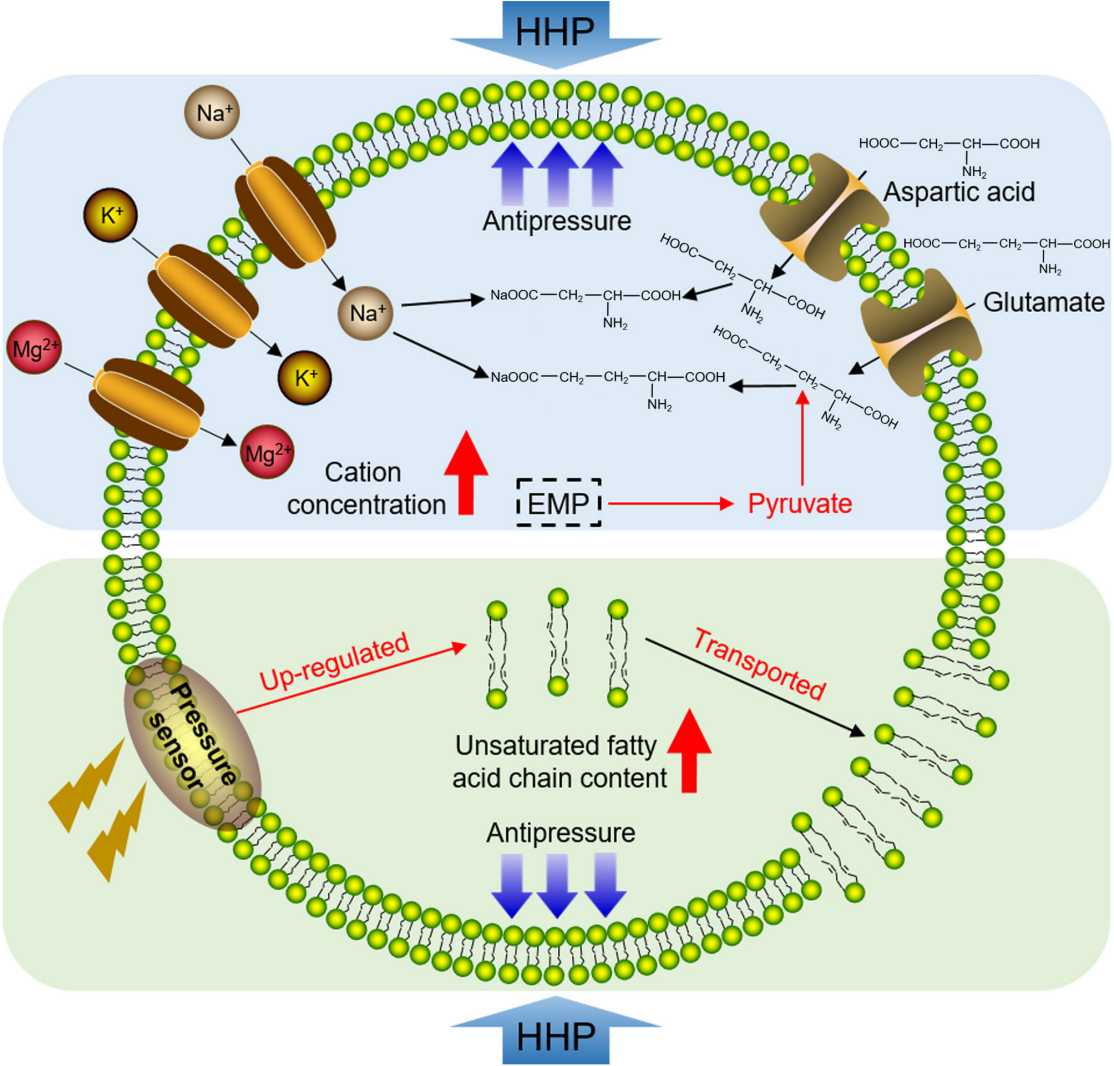
A proposed model of HHP tolerance in *H. nucleasis* zrk29. Strain zrk29 mainly tolerates HHP via two pathways: (i) by transporting cations into its cells to increase intracellular osmotic pressure and (ii) by adjusting unsaturated fatty acid chain content in its cell membrane phospholipids to increase cell membrane fluidity.

Our metabolomics assays also showed that the proportion of membrane phospholipids containing unsaturated fatty acid chains in cells from strains of zrk29 cultured under 12 MPa was significantly higher (by approximately two to three times) than in cells from strains cultured under 0.1 MPa (Fig. 5F). In addition, the relative abundances of different phospholipids containing unsaturated fatty acid chains, including phosphatidyl ethanolamines (PE), phosphatidyl glycerol (PG), phosphatidyl choline (PC), and phosphatidyl serine (PS) were higher in cells from strains of zrk29 cultured under 12 MPa than those cultured under 0.1 MPa by approximately 231, 22, 12, and 16 times, respectively (Fig. 5G). Furthermore, our fatty acid content determination assays showed that the contents of unsaturated fatty acids (C_16:1_ *cis*-9 FAME, C_18:1_ *cis*-9 FAME, and C_18:2_ *cis*-9,12 FAME) were much higher in the membrane lipids of strain zrk29 cultivated under 12 MPa than in the strain cultivated under 0.1 MPa (Fig. 5H). It can, therefore, be inferred that free-living wall-less strain zrk29 is able to tolerate HHP stress via two pathways (Fig. 6): (i) by transporting large amounts of cations into its cells to increase intracellular osmotic pressure and (ii) by increasing its phospholipid unsaturated fatty acid chain content to enhance cell membrane fluidity.

## DISCUSSION

Wall-less bacteria have evolved a broad range of lifestyles, including free-living, commensalism, and parasitism (28). Of particular interests are free-living *Izemoplasmatales* bacteria that actively participate in the primary degradation of extracellular DNA in deep marine sediments (5, 15, 16). To study the characteristics of previously uncultured bacteria, pure cultures are essential to enable detailed morphological and physiological characterization (29). However, the paucity of available isolates due to the culturing difficulties has greatly limited insights into free-living *Izemoplasmatales* bacteria. Here, we developed an innovative method to enrich samples and successfully cultivate the free-living wall-less strain zrk29 from the deep-sea sediment. This strain is the first representative of the novel family, *Hujiaoplasmataceae* (a sister group to the *Izemoplasmataceae* family), within the order *Izemoplasmatales* (Fig. 1A; Fig. S3 to S5; Table S1 to S7).

We have demonstrated for the first time that strain zrk29 has a strong ability to degrade nucleic acids (both DNA and RNA) (Fig. 2A and B), which is different from *Izemoplasmataceae* strain zrk13, which only degrades DNA (5). The number of genes encoding extracellular ribonucleases, endonucleases, and endoribonucleases in strain zrk29 is higher than in strain zrk13, consistent with strain zrk29’s strong RNA-degradation abilities (Data set S2). Therefore, members within the order *Izemoplasmatales* might have the ability to degrade different types of nucleic acids. As extracellular DNA is a major component of most bacteria (30), it is understandable that the culture supernatant of strain zrk29 inhibited the biofilm formation of *Pseudomonas aeruginosa* PAO1 (Fig. S10)—a notorious pathogenic bacterium with a strong ability to form biofilm. This unique ability of strain zrk29 has enabled it to adapt to harsh deep-sea conditions and may also help to prevent biofilm-related infection and pollution. Both the laboratory and *in situ* experimental results indicated that strain zrk29 could degrade and use nucleic acids to promote its growth using a variety of enzymes, particularly nucleases (Fig. 2). To understand the specific nucleic acid metabolism pathway of strain zrk29, we conducted in-depth analysis of its genome and found that it contained a complete set of nucleic acid metabolism genes (Fig. 3A). These nucleases (including extracellular ribonucleases, deoxyribonucleases, exonucleases, endonucleases, endoribonucleases, among others) degraded extracellular DNA and RNA into nucleotides; these were then degraded further into nucleosides and inorganic phosphates by 5′-nucleotidase. Subsequently, the purine and pyrimidine nucleosides were transported into the cell and entered the purine/pyrimidine nucleoside catabolic pathways where they were broken down further into different nucleobases by nucleoside phosphorylases. The metabolites of nucleoside catabolism were finally transformed into phosphoribosyl pyrophosphate and ribose-1P, which then entered into the oxidative pentose phosphate pathway—a pathway closely related to EMP glycolysis pathway. Ultimately, a considerable amount of energy was generated during nucleic acid metabolism, and this promoted strain zrk29 growth (Fig. 3B). Notably, despite a limited genome size, strain zrk29 maintains functional RM and CRISPR systems. CRISPR systems were also detected in the *Candidatus* Bathyoplasma sp. NZ (31) and 21 out of 52 wall-less species (32). This indicates that the effective immune system is used by many wall-less bacteria to protect themselves from viruses (33, 34), thereby enhancing their survival in the deep-sea environment (Fig. 3B).

Nucleic acids induced strain zrk29 to release the chronic bacteriophage Phage-zrk29 (Fig. 4A). Traditionally, most bacteriophages have either lytic or lysogenic lifecycles (35). Currently, the chronic infection cycle, characterized by continued bacterial growth despite phage reproduction (36), is increasingly capturing attention. In our study, the bacteriophages induced by the supplementation of nucleic acids showed no damaging effects on strain zrk29 growth, suggesting that these phages might use a chronic infection cycle. Moreover, we found many AMGs encoding nucleases present in Phage-zrk29, which may facilitate the host to metabolize and use extracellular nucleic acids in deep-sea environment (Fig. 4B). To verify whether the AMGs were expressed, we investigated the ability of Phage-zrk29 to facilitate nucleic acid metabolism and promote growth in other marine bacteria. We found that Phage-zrk29 significantly promoted the growth of the marine strain 273 by supporting its metabolism and utilization of nucleic acids (Fig. 4C and D). This provides stronger evidence that the relationships between Phage-zrk29 and its hosts are not characterized by a “fight to the death” between enemies but rather a mutualistic partnership (37).

The HHP of the deep sea was critical for the survival of wall-less bacteria. Adaptation mechanisms to HHP are varied among microorganisms from the deep biosphere, including osmolyte production, membrane adjustment, and transcription regulation (38). Previous reports showed the supplementation of Mg^2+^ increased the maximum growth pressure of a microbial community enriched from Mariana Trench sediment (39), while uptake of K^+^ has been observed in response to increased external osmolarity (40). The strong positively charged cations must be balanced to prevent damage to biological molecules and processes; gram-negative bacteria might therefore biosynthesize or acquire organic anions (such as glutamate) from the environment to counterbalance the accumulating cations (41 - 44). Consistently, we found the concentrations of cations (Mg^2+^, K^+^, and Na^+^) and organic anions (aspartic acid, glutamic acid, and glutamine) were all significantly upregulated under HHP (Fig. 5B through E), suggesting that *H. nucleasis* zrk29 responds to HHP by upregulating its intracellular osmotic pressure. Furthermore, the proportion of unsaturated fatty acid chains in phospholipids and the content of unsaturated fatty acid in strain zrk29 cultured under HHP were higher than in strains cultured under atmospheric pressure (Fig. 5F through H). Phospholipids (phosphatidyl ethanolamines, phosphatidyl glycerol, and phosphatidyl cardiolipin) are major constituents of cell membranes (45) and their fatty acid compositions are regulated to maintain membrane order and fluidity (46). Our study confirms that the free-living wall-less strain zrk29 tolerates HHP stress by increasing its intracellular osmotic pressure and increasing the unsaturated fatty acid chain content of phospholipids in its cell membrane (Fig. 6). Future research should focus more on free-living *Izemoplasmatales* bacteria to provide a deeper understanding of the mechanisms of adaptation to the extreme deep-sea environment.

In summary, our study into the first representative of *Hujiaoplasmataceae* (a novel family within the *Izemoplasmatales* order), under both laboratorial and deep-sea conditions extends our understanding of the mechanisms used by free-living wall-less bacteria to metabolize nucleic acids and to tolerate HHP. Our study also highlights that nucleic acids induce this free-living wall-less strain to release a chronic bacteriophage. This bacteriophage contains AMGs that encode nucleases, which may facilitate host metabolism, thereby contributing to oceanic and sedimentary biogeochemical cycling.

## MATERIALS AND METHODS

### Cultivation and phenotypic characterization of strain zrk29

Deep-sea samples were collected by *RV KEXUE* from a typical cold seep in the South China Sea (E119°17*'*07.322*''*, N22°06*'*58.598*''*) according to previously described methods (5, 47). To enrich the samples for free-living wall-less bacteria, they were cultivated at 28°C for 6 months in an anaerobic basal medium (1.0 g NH_4_Cl, 1.0 g NaHCO_3_, 1.0 g CH_3_COONa, 0.5 g KH_2_PO_4_, 0.2 g MgSO_4_·7H_2_O, 0.7 g cysteine hydrochloride, 500 µL 0.1% (wt/vol) resazurin in 1 L filtered seawater, pH = 7.0) supplemented with 100 µg/mL *E. coli* genomic DNA and 100 µg/mL RNA (48). Next, 50 µL of enrichment medium at various dilutions was spread onto a Hungate tube containing a basal medium supplemented with 100 µg/mL DNA, 100 µg/mL RNA, and 1.5 mg/mL agar. The tubes were then incubated at 28°C for 10 days. Individual colonies with distinct morphology were selected using sterilized bamboo sticks. The colonies were then cultured in a nutrient-rich medium (1.0 g yeast extract, 1.0 g peptone, 1.0 g NH_4_Cl, 1.0 g NaHCO_3_, 1.0 g CH_3_COONa, 0.5 g KH_2_PO_4_, 0.2 g MgSO_4_.7H_2_O, 0.7 g cysteine hydrochloride, 500 µL 0.1% (wt/vol) resazurin in 1 L filtered seawater, pH = 7.0) using the Hungate roll-tube methods for several cycles until they were considered to be pure. The purity of free-living wall-less bacterium zrk29 was confirmed by TEM followed by repeated partial sequencing of the 16S rRNA gene. The pure isolate was then preserved at −80°C with 20% (vol/vol) glycerol.

### TEM observation

To observe the morphological characteristics of strain zrk29, the cell suspension was washed with Milli-Q water and centrifuged at 5,000 × *g* for 20 minutes. Cells were then collected by immersing copper grids coated with a carbon ﬁlm in the cell suspension for 20 minutes. The grids were then washed for 10 minutes in distilled water and dried for 20 minutes at room temperature (5). For ultrastructure observation, ultrathin-section electron microscopy was performed, as described previously (49). Briefly, cultures of strain zrk29 were grown to a maximum density in a rich medium and then centrifuged at 5,000 × *g* for 10 minutes. The samples were initially preserved in 2.5% (vol/vol) glutaraldehyde overnight at 4°C. They were then washed three times with PBS and dehydrated in ethanol solutions of 30%, 50%, 70%, 90%, and 100% for 10 minutes each. The samples were then embedded in a plastic resin. Ultrathin sections (approximately 50–70 nm) of cells were prepared using an ultramicrotome (Leica EM UC7, Germany) and then stained with uranyl acetate and lead citrate. All samples were examined using a TEM (HT7700, Hitachi, Japan) with a JEOL JEM 1200 EX (equipped with a ﬁeld emission gun) at 100 kV (5).

### Phylogenetic analysis

For the phylogenetic analysis, the full-length 16S rRNA gene sequence of strain zrk29 was obtained from its genome and other related taxa from NCBI (www.ncbi.nlm.nih.gov/). The genome-based phylogenetic tree was reconstructed using the GTDB Toolkit (https://github.com/Ecogenomics/GTDBTk) based on the concatenated alignment of 120 ubiquitous single-copy proteins (50). The RpoB and EF-tu trees were constructed based on RpoB or EF-tu protein sequences, which were identified from 174 genomes using the hidden Markov models (HMMs) TIGR02029 and TIGR00485 from TIGRfams (http://www.jcvi.org/cgi-bin/tigrfams/index.cgi), respectively (15). All the sequences were aligned by MAFFT version 7 (51) and manually corrected. The phylogenetic trees were then constructed using the W-IQ-TREE web server (http://iqtree.cibiv.univie.ac.at) (52) with the LG + F + I + G4 model. The Interactive Tree of Life (iTOL version 5) online tool (53) was then used to edit the phylogenetic trees.

### Detection of DNA and RNA degradation by *H. nucleasis* zrk29

As described previously (5), agarose gel electrophoresis was performed to detect nucleic acid degradation by strain zrk29. The *Escherichia coli* genomic DNA and RNA were, respectively, extracted from *E. coli* DH5α (Tsingke, China) cells using the genomic DNA and RNA Extraction Kits (Tsingke, China) according to the manufacturer’s instructions. The reaction system (10 µL) for this assay contained 4 µL *E. coli* genomic DNA (1.5 µg), 4 µL *E. coli* RNA (1.0 µg), 1 µL bacterial spent culture, and 1 µL FastDigest Green Buffer (10×) (Thermo Fisher Scientific, USA). This assay used two control groups: *E. coli* genomic DNA and RNA degraded by the medium without cells and the culture supernatant of a *Clostridia* bacterium. The *E. coli* genomic DNA and RNA degraded by the culture supernatant of strain zrk29 were used as the experimental group. The culture supernatant was obtained by centrifugation with 12,000 × *g* for 10 minutes. The reactions were conducted either at 37°C for 5 , 15, and 30 minutes or at 4°C for 1, 2, and 3 hours. Finally, the presence of *E. coli* genomic DNA and RNA in the reaction solutions was detected using 1% agarose gel electrophoresis, run at 180 V for 15 minutes; the concentrations were measured by Nanodrop (IMPLEN, Germany), and gel images were taken using the Gel Image System (Tanon 2500, China).

### Growth assay and transcriptomic analysis of *H. nucleasis* zrk29 incubated in the medium supplemented with nucleic acids

To assess the effect of nucleic acids on the growth of strain zrk29, 30 mL of freshly incubated cells was inoculated in 1.5 L of either basal medium alone, basal medium supplemented with 100 µg/mL *E. coli* genomic DNA, and 100 µg/mL RNA, or rich medium at 28°C for 15 days. As described previously (5), since strain zrk29 grew slowly in the basal medium, quantitative real-time PCR (qRT-PCR) was used to measure its growth curve. Correspondingly, a 100 mL culture was harvested daily and subsequently centrifuged at 8,000 × *g* for 20 minutes, which was then used for qRT-PCR. Specific primers for 16S rRNA genes (Table S8) and genes relating to nucleic acid degradation (Table S9) were designed using Primer 5.0. For transcriptomic analysis, cell suspensions of strain zrk29 were cultured in 1.5 L of either basal medium alone or basal medium supplemented with 100 µg/mL *E. coli* genomic DNA and 100 µg/mL RNA for 7 days. The cells were then collected by centrifuging at 8,000 × *g* for 20 minutes. The transcriptomic sequencing was performed by Novogene (Tianjin, China). Detailed protocols of the procedures used, including library preparation, clustering, and sequencing, and data analyses were described in the supplementary information.

### Transcriptomic analysis of the *H. nucleasis* zrk29 strain incubated in a deep-sea cold seep

To explore the metabolism of strain zrk29 in the deep-sea cold seep, the strain was initially cultured in a rich medium for 7 days. Following this, 30 mL of the fresh cultures was then transferred to 1.5 L of the rich medium. Thereafter, the culture medium of strain zrk29 was divided into two parts: one part was divided and transferred equally into three gas sample bags (which not allowing any exchanges between inside and outside; aluminum-plastic composite film, Hede, China) with 200 mL culture medium each and set as control groups; the other part was divided into three dialysis bags (8,000–14,000 Da cutoff, which allowing the exchanges of substances smaller than 8,000 Da but preventing bacterial cells from entering or leaving the bag; Solarbio, China) with 200 mL culture medium each and set as experimental groups. All the samples were placed simultaneously in the deep-sea cold seep (E119°17′04.429″, N22°07′01.523″) for 10 days in June 2020 during the cruise of *Kexue* vessel. After 10 days of *in situ* incubation, the bags were recycled, and the cells were immediately collected and kept at −80°C for future analysis. The cells were checked by 16S rRNA sequencing to confirm the purity of the cultures and subsequently investigated further by transcriptomics analysis. The total RNA of strain zrk29 was then extracted using TRIzol reagent (Invitrogen, USA). DNA contamination was removed using the MEGA clear Kit (Life technologies, USA). The detailed protocols for transcriptomic sequencing were conducted as described above.

### Bacteriophage isolation

Phage isolation experiments were performed according to previously described methods (54 - 56). To isolate the bacteriophages, strain zrk29 was inoculated in either rich medium alone or rich medium supplemented with 100 µg/mL *E. coli* genomic DNA and RNA for 14 days. The culture supernatant was collected by centrifugation at 8,000 × *g* at 4°C for 20 minutes and then filtered through a 0.22-µm millipore filter (Pall Supor, New York, America). An amount of 1 M NaCl was then added to the filtered supernatant and precipitated on the ice for 2 hours to further remove the residual bacterial fragments. The supernatant was then collected by centrifugation at 8,000 × *g* at 4°C for 20 minutes. The phage particles were immediately precipitated from the supernatant using 100 g/L polyethylene glycol (PEG 8000) at 4°C for 6 hours, and then collected by centrifugation at 10,000 × *g* at 4°C for 30 minutes. The phage particles were suspended in 2 mL SM buffer (0.01% gelatin, 50 mM Tris-HCl, 100 mM NaCl, and 10 mM MgSO_4_) and then extracted three times using equal volumes of chloroform (57). Finally, the clean phage particles were collected by centrifugation at 4,000 × *g* at 4°C for 20 minutes.

### Growth assay of *Pseudomonas stutzeri* 273 cultured in an inorganic medium supplemented with nucleic acids and Phage-zrk29

To test the ability of the bacteriophages induced by strain zrk29 to assist host metabolism and utilization of nucleic acids, an aerobic *P. stutzeri* 273 was selected and isolated from a deep-sea sediment (27). Specifically, 50 µL of freshly incubated cells (*P. stutzeri* 273) was inoculated in 5 mL of inorganic medium (10 g/L NaCl, 1 L sterilized distilled water); inorganic medium supplemented with 20 µL/L Phage-zrk29; inorganic medium supplemented with 100 µg/mL *E. coli* genomic DNA and 100 µg/mL RNA; or basal medium supplemented with 100 µg/mL *E. coli* genomic DNA, 100 µg/mL RNA, and 20 µL/L Phage-zrk29 for 24 hours at 28°C. Three replicates were performed for each condition. Bacterial growth was monitored by measuring OD_600_ values via a microplate reader (Infinite M1000 Pro; Tecan, Mannedorf, Switzerland) every 4 hours until cell growth reached a stationary phase.

### Growth assays, transcriptomic, and metabolomic analyses of *H. nucleasis* zrk29 cultured under different pressures

To test the effect of HHP on strain zrk29 growth, we cultured and monitored its growth under 12 MPa of pressure, similar to the pressure of the deep-sea cold seep where strain zrk29 was isolated from. Briefly, 15 mL of freshly cultured cells from the corresponding strains was inoculated in a sterile anaerobic bag (Hede, Dalian, China) containing 0.75 L rich medium; six replicates were performed. Three bags were placed inside the pin closure pressure vessel (Feiyu Petrochemical Instrument Equipment Inc., Nantong, China) with an HHP of 12 MPa for 7 days at 28°C. These strains acted as the test groups. The other three anaerobic bags were cultured at atmospheric pressure (0.1 MPa) for 7 days at 28°C and acted as the control groups. After incubation, the cell suspensions were collected by centrifuging at 8,000 × *g* for 20 minutes. Finally, qRT-PCR was performed to measure strain zrk29 growth under different pressures. The primers used are shown in Table S8.

To understand the mechanisms strain zrk29 uses to tolerate HHP, cell suspensions of strain zrk29 were cultured under 0.1 MPa and 12 MPa, as described above. Further transcriptomic and metabolomic analyses were then performed by Novogene (Tianjin, China). The procedures used for the transcriptomic and metabolomic analyses are described in the supplementary information.

### Measurement of intracellular cation concentrations and composition ratios of membrane lipids of *H. nucleasis* zrk29 cultured under different pressures

To measure the concentrations of Mg^2+^, K^+^, and Na^+^ in strains of zrk29 cultured under 0.1 MPa and 12 MPa for 7 days, cell suspensions were collected by centrifuging at 8,000 × *g* for 20 minutes. The intracellular concentrations of different cations of strain zrk29 were then measured by the company, YuanCe, in Qingdao, China. Briefly, 0.25 g of the cell sample was weighed out and placed in a high-temperature-resistant and pressure-resistant microwave digestion tank with 5 mL of nitric acid. After digestion was complete, the solution was transferred to a 25-mL volumetric flask, and 200-µL metal element standard solution (1 µg/mL) was added. The solution was then diluted with water to the required scale. Finally, the prepared sample was checked using the Agilent 5110 ICP-OES (NYSE: A, Palo Alto, USA). The proportion of membrane lipids containing unsaturated fatty acid chains in strain zrk29 cultured under 0.1 MPa and 12 MPa was then tested by metabolomic analysis (described above). Cellular fatty acids were extracted from dried cells and analyzed by gas chromatography (model 7890A, Agilent) to determine their composition. This was performed according to the protocol of the Sherlock Microbial Identification System.

### Description of ***Hujiaoplasma*** gen. nov

*Hujiaoplasma* (Hu.jiao'plas.ma. N.L. fem. pl. n. *Hujiao* comes from a strange animal documented in the famous Chinese myth, “Classic of Mountains and Rivers”; -plasma, formed or molded, refers to the lack of a cell wall) are wall-less, free-living, non-motile, and non-spore forming coccoid cells. Yeast extract and acetate can promote its growth. Its phylogenetic position is in the family *Hujiaoplasmataceae*, order *Izemoplasmatales*, class *Bacilli* of the phylum *Firmicutes*. The type species is *Hujiaoplasma nucleasis*.

### Description of *Hujiaoplasma nucleasis* sp. nov

*Hujiaoplasma nucleasis* (nu.clea′sis. L. gen. pl. n. *nucleasis* implies a strong ability to degrade nucleic acids).

Cells are coccoid, obligately anaerobic, neutrophilic, mesophilic, moderately halophilic, and had no flagellum. Most cells have a diameter of 300–500 nm. Grow at optimum pH values of 7.0. The optimum temperature range for growth is 28–30°C. Growth occurs at NaCl optimum concentrations between 0.0% and 4.0%. From the sole carbon source utilization test, growth is stimulated by glucose, maltose, fructose, sucrose, acetate, formate, isomaltose, lactate, D-mannose, glycerin, and rhamnose.

The type strain, zrk29^T^ (CGMCC = 1.17882), was isolated from the sediment of deep-sea cold seep, P.R. China. The DNA G + C content of the type strain is 30.68%.

### Description of *Hujiaoplasmataceae* fam. nov

*Hujiaoplasmataceae* (Hu.jiao'plas.ma.taceae N.L. fem. n. *Hujiaoplasma*, type genus of the family; suff. -*taceae*, ending to denote a family; N.L. fem. pl. n. *Hujiaoplasmataceae*, the *Hujiaoplasma* family). The description is the same as that for the genus *Hujiaoplasma*. The type genus is *Hujiaoplasma*.

## Data Availability

The complete genome sequence of strain zrk29 presented in this study has been deposited in the GenBank database with accession number CP051151. The whole 16S rRNA sequence of strain zrk29 has been deposited in the GenBank database with accession number MT793108. The raw sequencing reads from the transcriptomics analysis have been deposited to the NCBI Short Read Archive (accession numbers: PRJNA672795, PRJNA750283, and PRJNA751724). The raw sequencing reads from the metabolomics analysis have been deposited to NCBI Short Read Archive (accession number: PRJNA754699). The genome sequence of Phage-zrk29 has been deposited in the GenBank database with accession number OP650936.

## SUPPLEMENTAL MATERIAL

Supplemental Figures mbio.00958-23-s0001.docx Fig. S1-S10.

Supplemental Text mbio.00958-23-s0002.docx Supplemental methods.

Supplemental Data Sets mbio.00958-23-s0003.xlsx Data Sets S1 and S2.

Supplemental Tables mbio.00958-23-s0004.docx Tables S1-S9.

ASM does not own the copyrights to Supplemental Material that may be linked to, or accessed through, an article. The authors have granted ASM a non-exclusive, world-wide license to publish the Supplemental Material files. Please contact the corresponding author directly for reuse.
