# Supplementary material for: Mechanisms of nucleic acid degradation and high hydrostatic pressure tolerance of a novel deep-sea wall-less bacterium
Source: mBio. 2023 Aug 8;14(4):e00958-23. doi: 10.1128/mbio.00958-23 (PMC10470597; doi:10.1128/mbio.00958-23)
Supplement: Supplemental Figures — Fig. S1-S10. [file mbio.00958-23-s0001.docx]

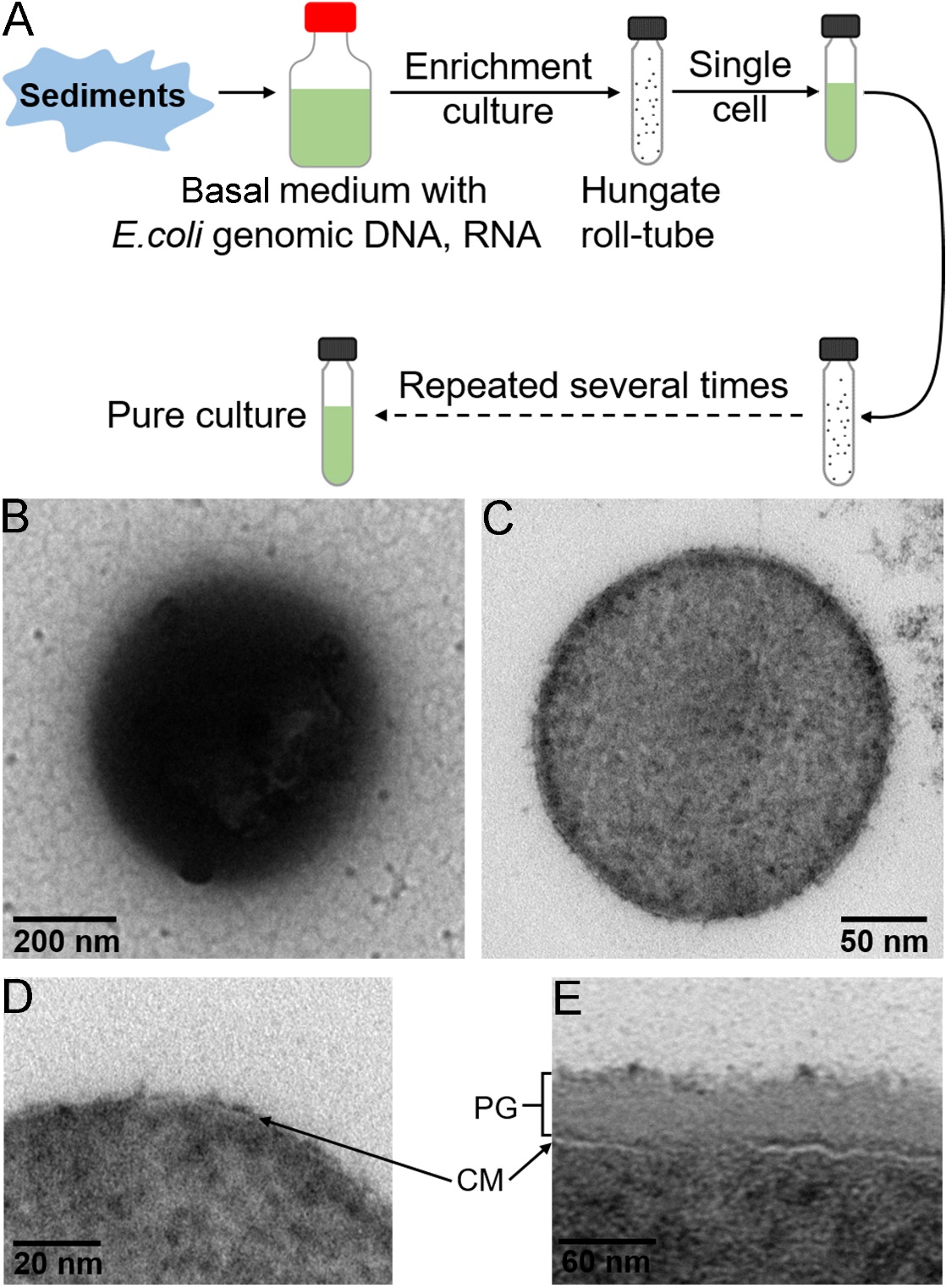


**FIG S1.** Isolation strategy and morphology of strain zrk29. (A) Schematic diagram of the isolation and purification process of strain zrk29. (B and C) Transmission electron microscopy (TEM) observation of strain zrk29. (D) Ultrathin sections observation of strain zrk29. (E) Gram-positive bacterial cell wall: *Bacillus* sp. zrkA. CM, cell membrane; PG, peptidoglycan.


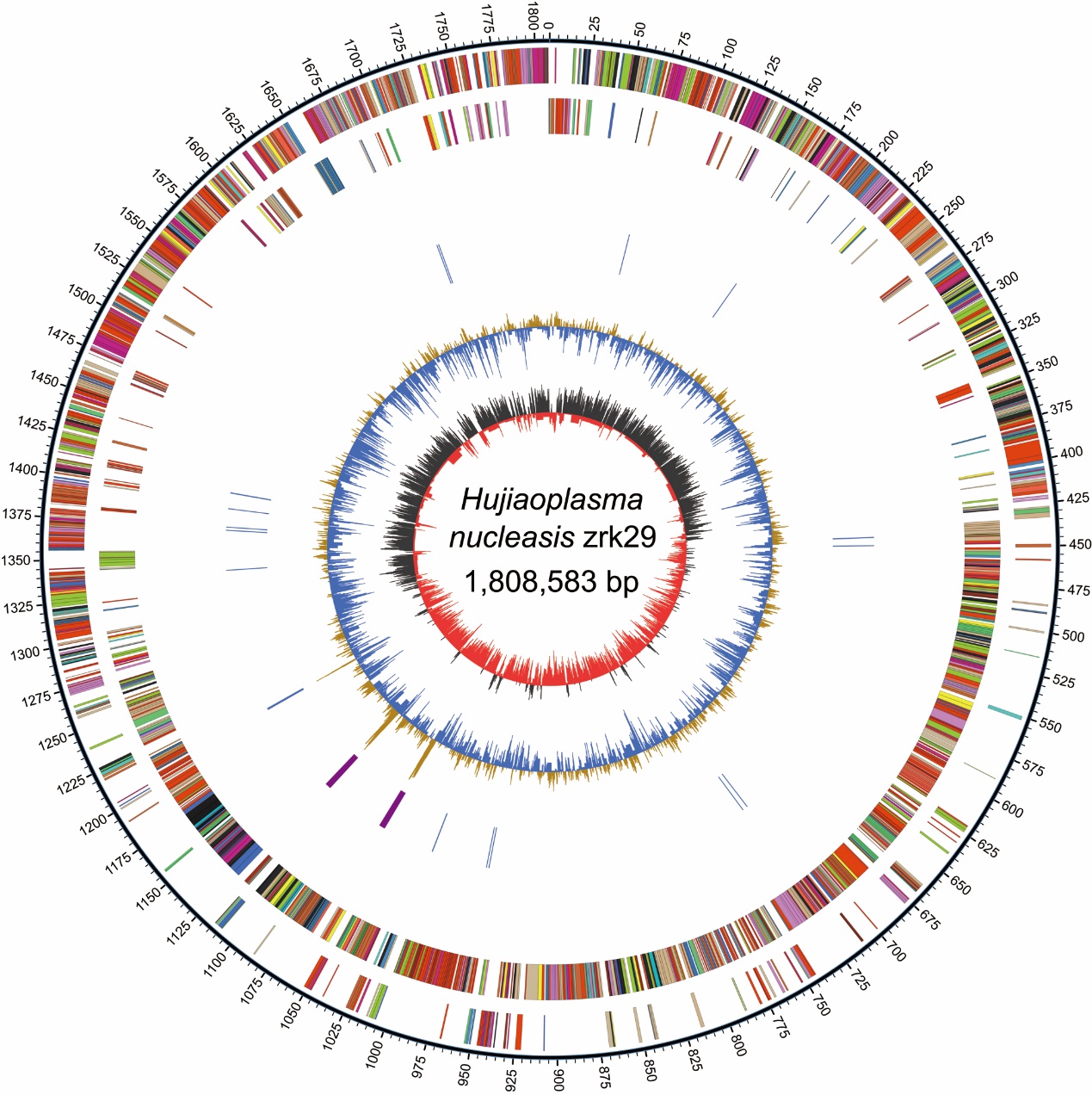


**FIG S2.** Circular diagram of strain zrk29 genome. Rings indicate, from outside to the center: A genome-wide marker with a scale of 25 kb; Forward strand genes, colored by COG category; reverse strand genes, colored by COG category; Repetitive sequences; RNA genes (tRNAs blue, rRNAs purple); GC content; GC skew.


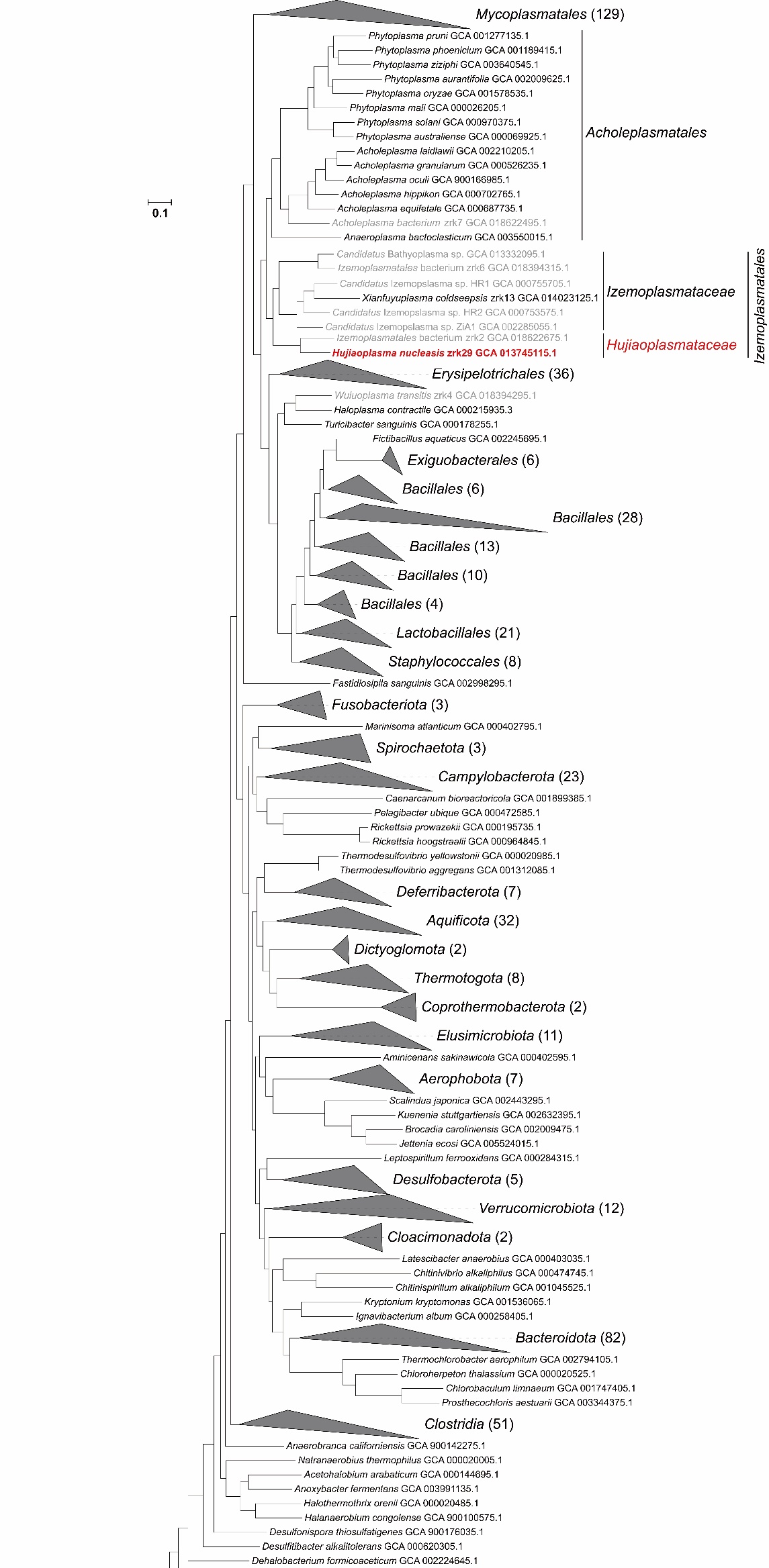


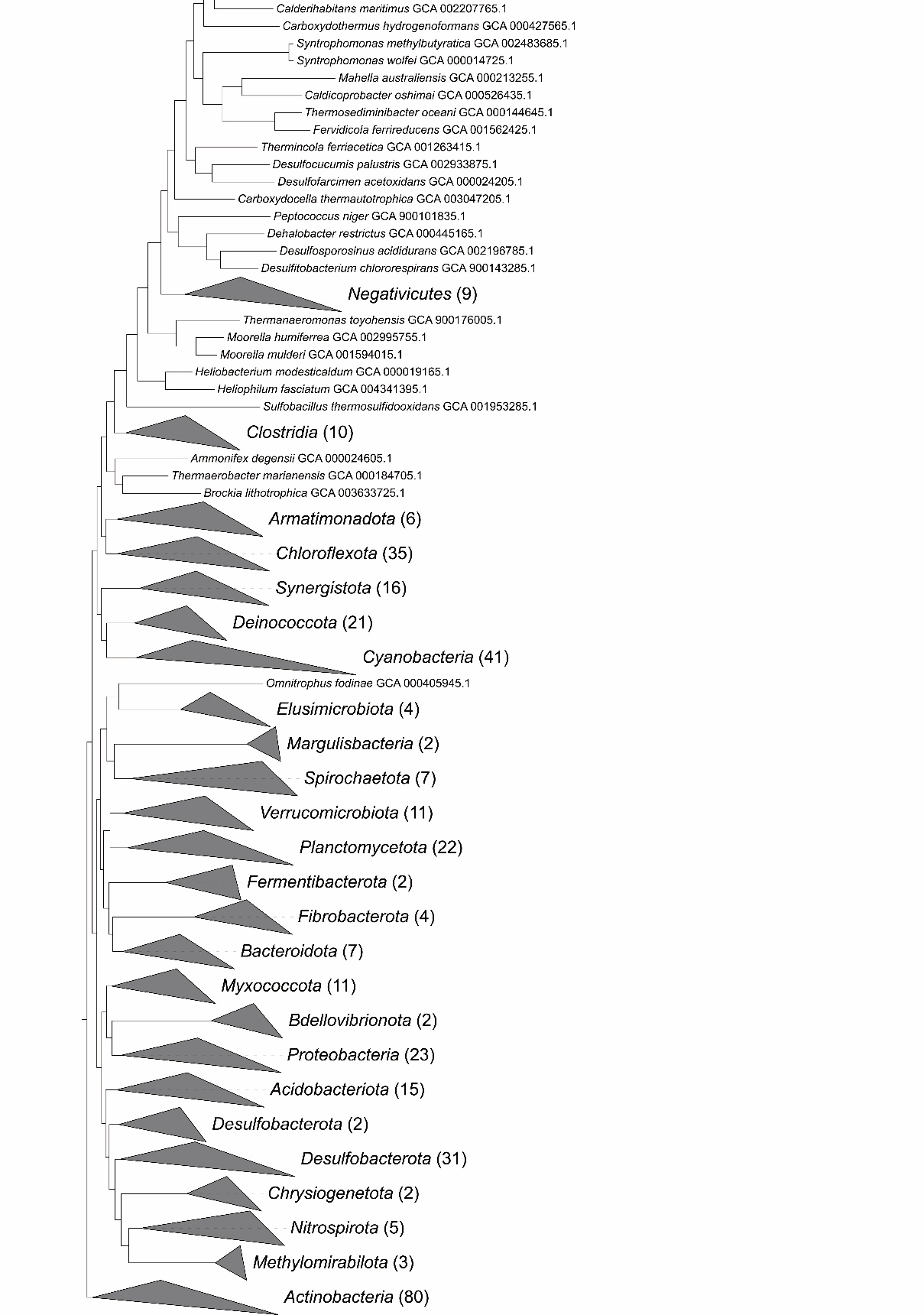


**FIG S3.** Maximum likelihood phylogenetic tree of strain zrk29 was reconstructed using the Genome Taxonomy Database Toolkit (https://github.com/Ecogenomics/GTDBTk) based on the concatenated alignment of 120 ubiquitous single-copy proteins. Some *Actinobacteria* members were used as the outgroup. Bar, 0.1 substitutions per nucleotide position.


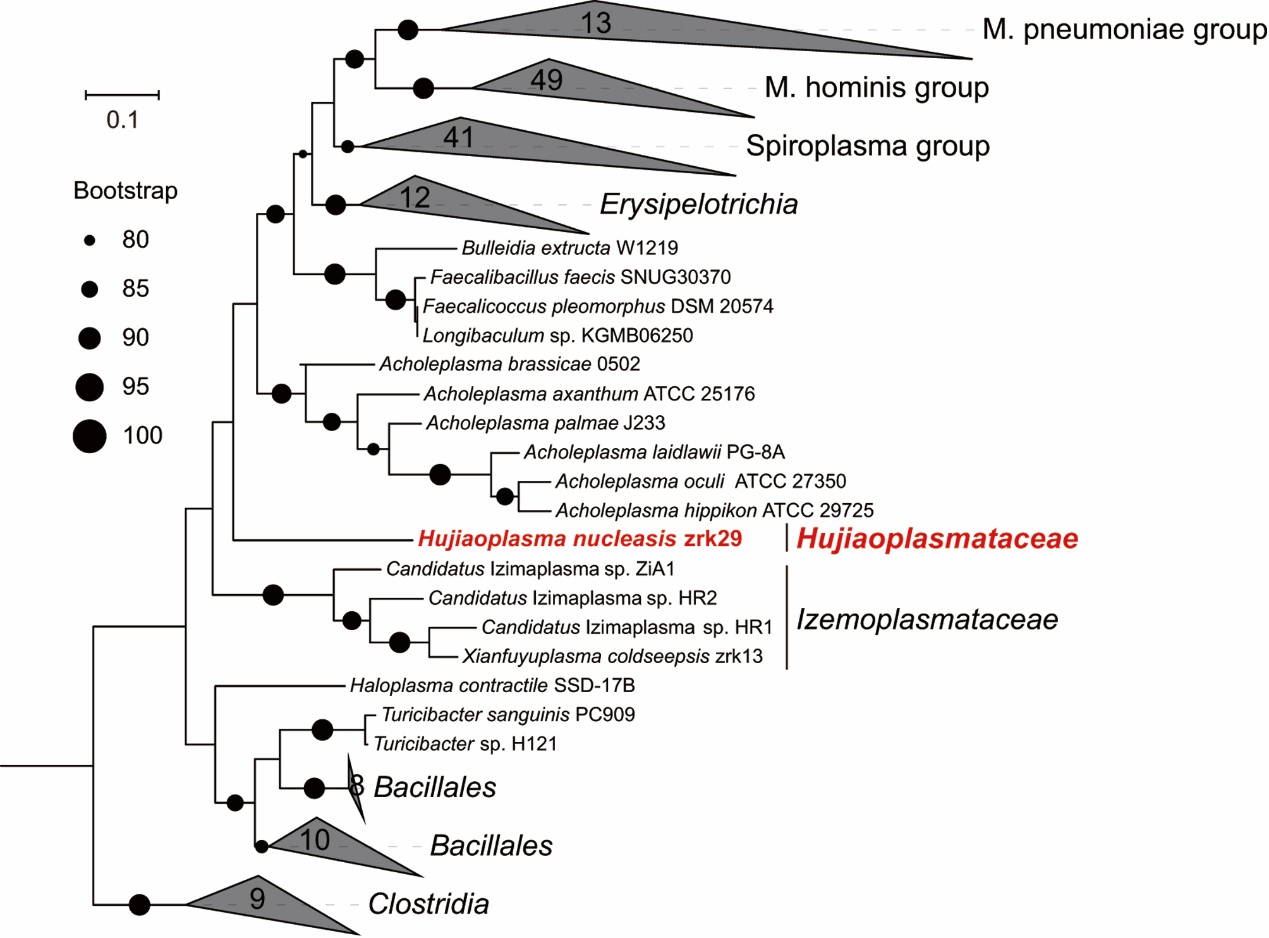


**FIG S4.** Maximum likelihood phylogenetic tree of elongation factor Tu (EF-Tu) from genomes of strain zrk29, other wall-less bacteria, and *Bacillales* representatives. The class *Clostridia* was used as the outgroup. Collapsed wedges represent monophyletic groups of EF-Tu. Nodes with greater than 80% bootstrap support are annotated with a black circle. Bar, 0.1 substitutions per nucleotide position.


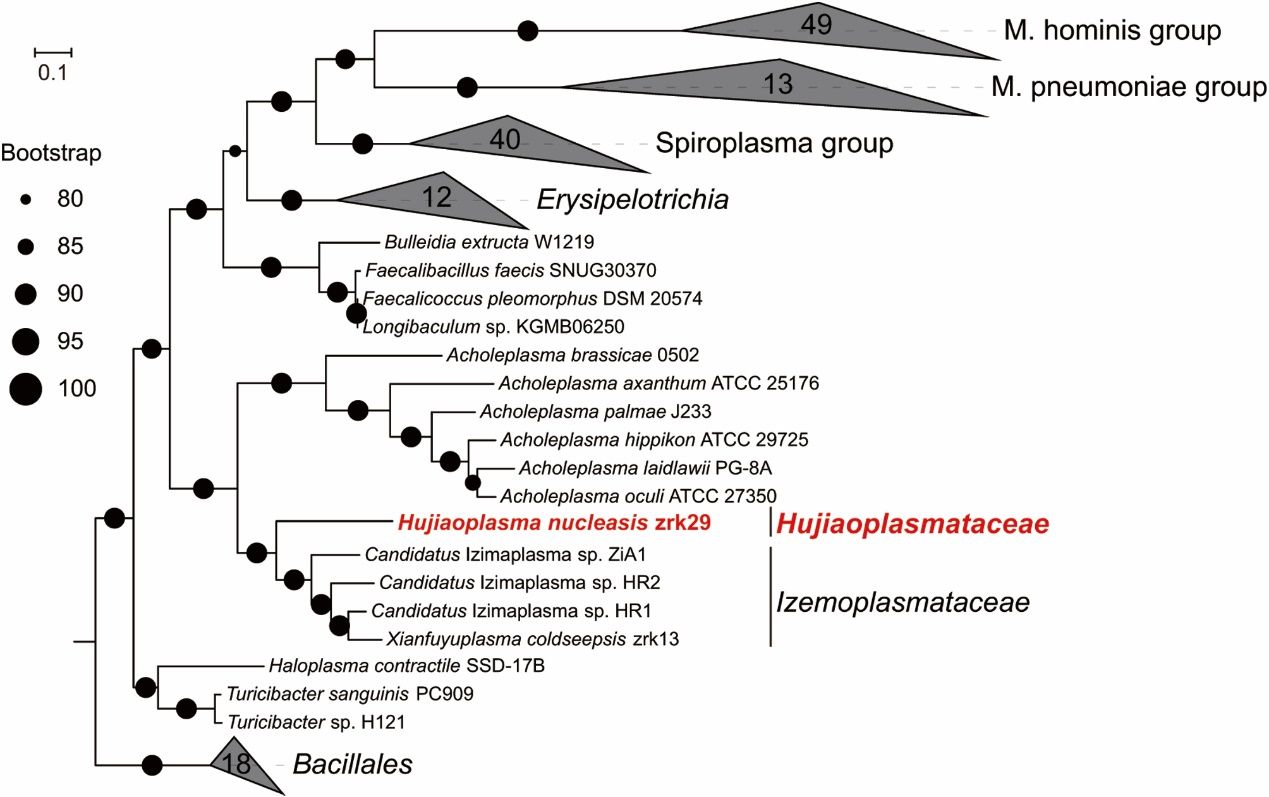


**FIG S5.** Maximum likelihood phylogenetic tree of RpoB from genomes of strain zrk29, other wall-less bacteria, and *Bacillales* representatives. The order *Bacillales* was used as the outgroup. Collapsed wedges represent monophyletic groups of RpoB. Nodes with greater than 80% bootstrap support are annotated with a black circle. Bar, 0.1 substitutions per nucleotide position.


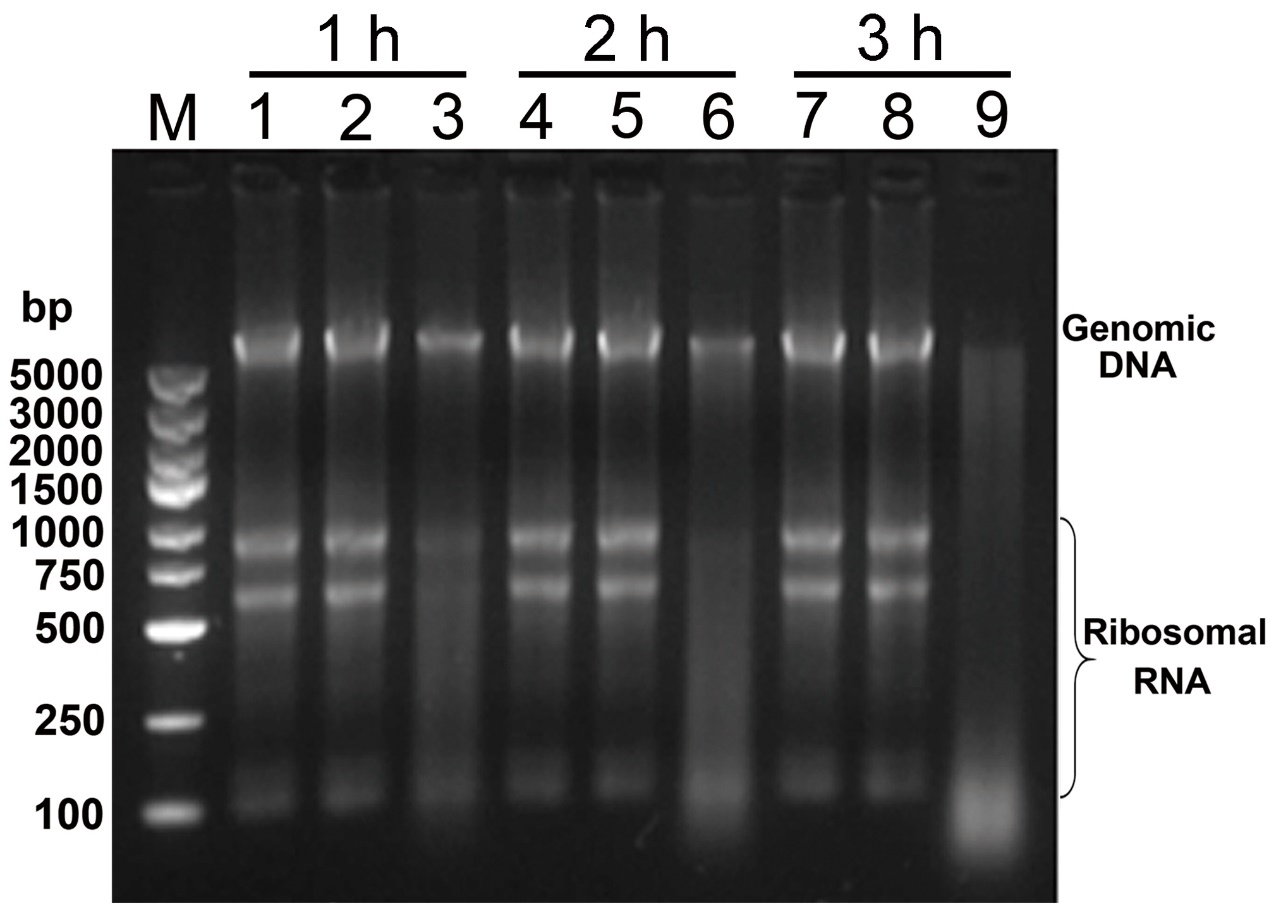


**FIG S6.** DNA and RNA degradative ability of strain zrk29 at 4 °C, visualized by agarose gel electrophoresis. Lane 1, lane 4, and lane 7 indicate 1.5 µg of *E. coli* genomic DNA and 1.0 µg of RNA treated with medium without cells for 1 hour, 2 hours, and 3 hours at 4 °C, respectively. Lane 2, lane 5, and lane 8 indicate 1.5 µg of *E. coli* genomic DNA and 1.0 µg of RNA treated with the supernatant of a *Clostridia* bacterium for 1 hour, 2 hours, and 3 hours at 4 °C, respectively. Lane 3, lane 6, and lane 9 indicate 1.5 µg of *E. coli* genomic DNA and 1.0 µg of RNA treated with the supernatant of strain zrk29 for 1 hour, 2 hours, and 3 hours at 4 °C, respectively. M, DL5000 molecular weight DNA marker.


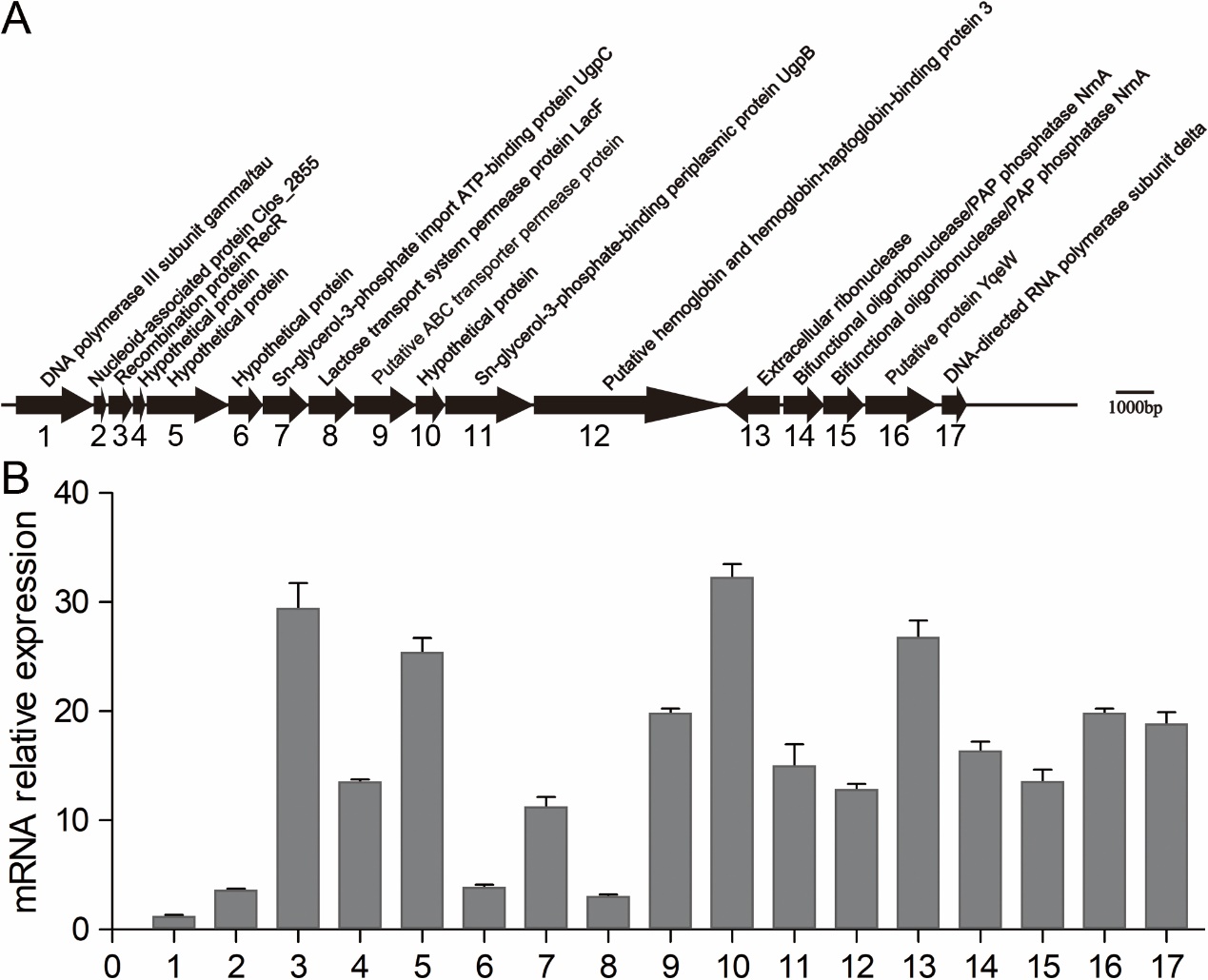


**FIG S7.** A complete cluster of nucleic acid-degrading genes in strain zrk29. (A) Gene arrangement of a putative nucleic acid-degradation locus in strain zrk29. The numbers shown in the *X*-axis indicate the code names of different genes within the gene cluster. (B) qRT-PCR detection of expression changes of genes shown in panel A when strain zrk29 was cultivated in basal medium supplemented either with or without 100 µg/ml *E. coli* genomic DNA and 100 µg/ml RNA. Three biological replicates were performed. The code numbers shown in the *X*-axis indicate the gene names shown in panel A.


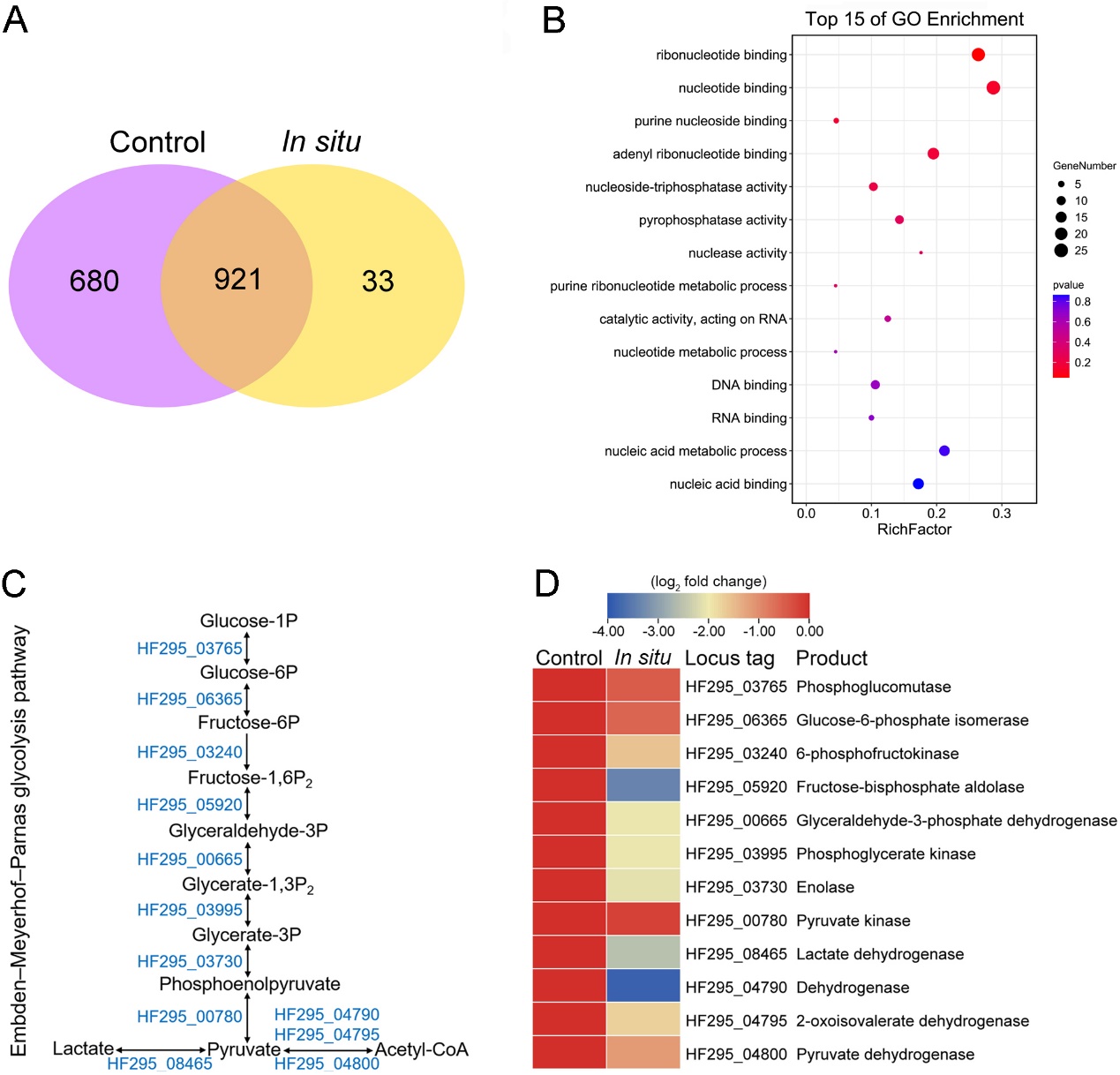


**FIG S8.** Transcriptomic analysis of strain zrk29 incubated in a deep-sea cold seep. (A) Differentially expressed genes between *in situ* and control groups based on transcriptomic analysis of strain zrk29 incubated in the deep-sea cold seep for ten days. (B) GO enrichment showing upregulated genes associated with nucleic acid degradation after a ten-day incubation of strain zrk29 in a deep-sea cold seep. (C) Diagrammatic scheme of Embden-Meyerhof-Parnas (EMP) glycolysis pathway. The gene numbers showing in this scheme are the same with those shown in panel D. (D) Transcriptomics-based heat map showing all downregulated genes associated with EMP glycolysis pathway after a ten-day incubation of strain zrk29 in the deep-sea cold seep environment.


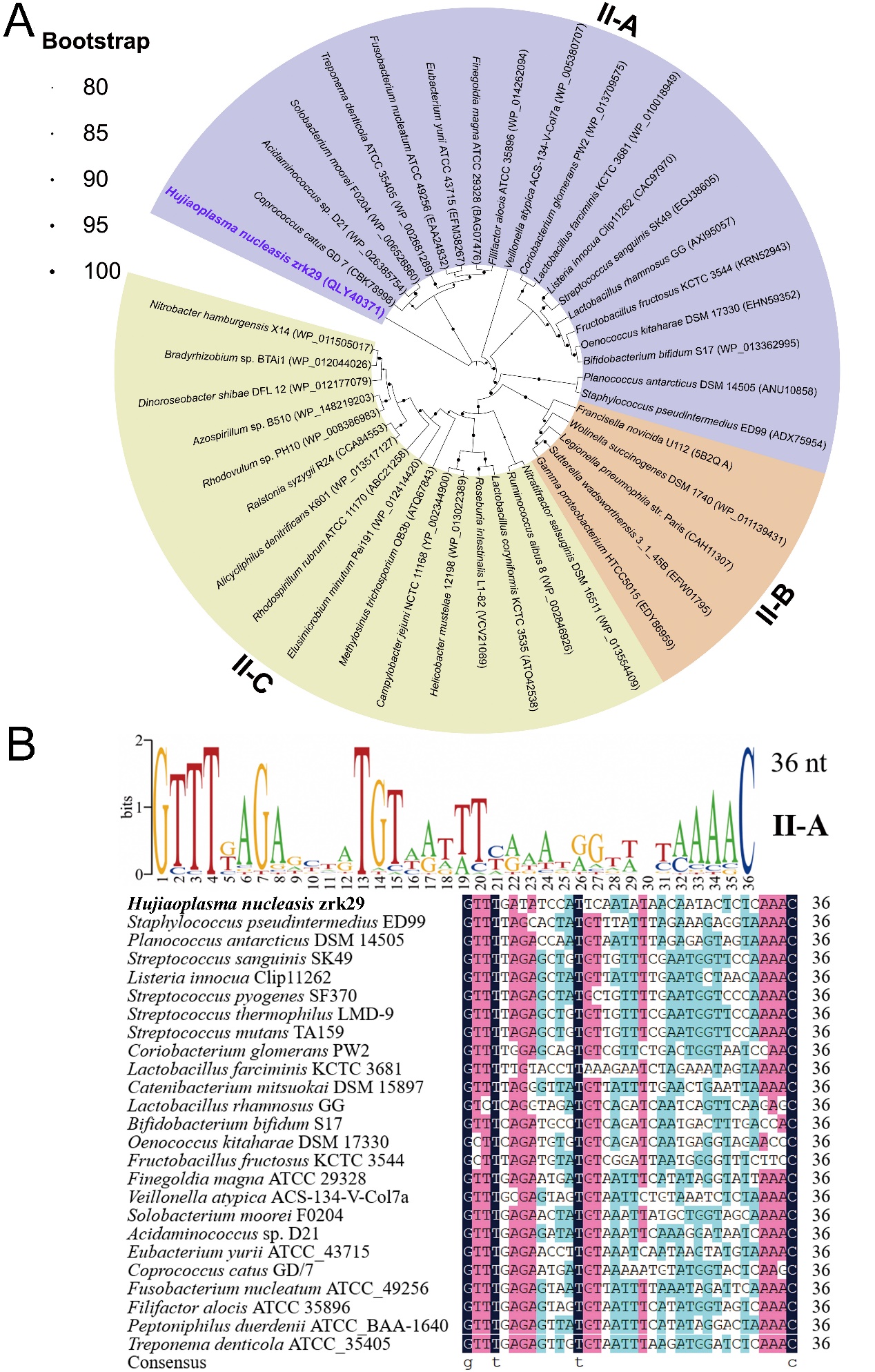


**FIG S9.** Alignment analysis of CRISPR system in strain zrk29. (A) Cas9 phylogeny as a basis for type II CRISPR system classification. The tree is inferred and reconstructed under the maximum likelihood criterion. Nodes with greater than 80% bootstrap support (expressed as percentages of 1,000 replications) are annotated with a black circle. (B) Multiple alignments of strain zrk29 and other type II-A associated CRISPR repeats. A multiple alignment for a representative set of CRISPR repeats was constructed using the MUSCLE program.


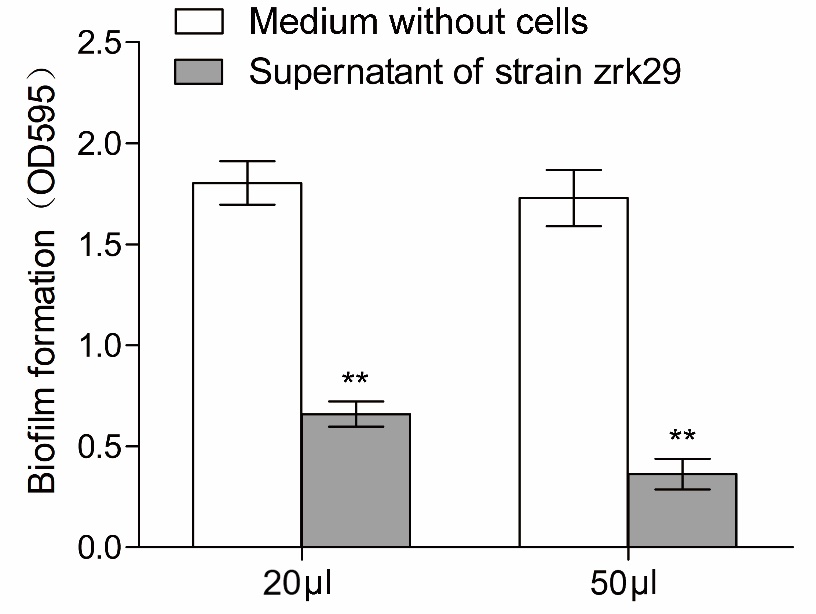


**FIG S10.** Supernatant of *H. nucleasis* zrk29 (20 µl or 50 µl) significantly inhibits biofilm formation of *Pseudomonas aeruginosa* PAO1.
