# Supplementary material for: Mechanisms of nucleic acid degradation and high hydrostatic pressure tolerance of a novel deep-sea wall-less bacterium
Source: mBio. 2023 Aug 8;14(4):e00958-23. doi: 10.1128/mbio.00958-23 (PMC10470597; doi:10.1128/mbio.00958-23)
Supplement: Supplemental Text — Supplemental methods. [file mbio.00958-23-s0002.docx]

**Supplementary Methods**

**Mechanisms of nucleic acid degradation and high hydrostatic pressure tolerance of a novel deep-sea wall-less bacterium**

Rikuan Zheng^1,2,4^, Chong Wang^1,2,4^, Ruining Cai^1,2,3,4^, Yeqi Shan^1,2,3,4^, Chaomin Sun^1,2,3,4*^

^1^CAS and Shandong Province Key Laboratory of Experimental Marine Biology & Center of Deep Sea Research, Institute of Oceanology, Chinese Academy of Sciences, Qingdao, China.

^2^Laboratory for Marine Biology and Biotechnology, Qingdao National Laboratory for Marine Science and Technology, Qingdao, China.

^3^College of Earth Science, University of Chinese Academy of Sciences, Beijing, China.

^4^Center of Ocean Mega-Science, Chinese Academy of Sciences, Qingdao, China.

^*^ Corresponding author

Chaomin Sun Tel.: +86 532 82898857; fax: +86 532 82898857.

E-mail address: sunchaomin@qdio.ac.cn

**SUPPLEMENTARY METHODS**

**Phenotypic characteristics of *H. nucleasis* zrk29.** For phenotypic characteristics, strain zrk29 was grown on rich medium (including 1.0 g yeast extract, 1.0 g peptone, 1.0 g NH_4_Cl, 1.0 g NaHCO_3_, 1.0 g CH_3_COONa, 0.5 g KH_2_PO_4_, 0.2 g MgSO_4_**^.^**7H_2_O, 0.7 g cysteine hydrochloride, 500 µl 0.1 % (w/v) resazurin in 1 L filtered seawater, pH=7.0). The temperature, pH, and NaCl concentration ranges for growth assays were determined in liquid rich-medium. Growth assay was performed at different temperatures (4, 16, 28, 30, 37, 45, 60, 70, 80 °C) for 14 days. The pH range for growth was tested in liquid rich medium from pH 4.0 to pH 10.0 at 28 °C for 14 days. Salt tolerance was tested on modified liquid rich medium (replaced sea water with distilled water) supplemented with 0-10 % (w/v) NaCl (0.5% intervals) at 28 °C for 14 days. Substrates utilization was tested in the medium (consisting of 5.0 g/l NaCl, 1.0 g/l NH_4_Cl, 0.5 g/l KH_2_PO_4_, 0.2 g/l MgSO_4_, 0.02 g/l yeast extract, pH 7.0) respectively supplemented with 20 mM single substrate (including glucose, maltose, butyrate, fructose, sucrose, acetate, formate, isomaltose, trehalose, galactose, xylose, lactate, D-mannose, glycerin, rhamnose, and sorbitol). Cell culture containing only 0.02 g/l yeast extract without adding any other substrates was used as a control. All the cultures were incubated at 28 °C for 14 days. Three replicates were performed.

**Genomic characterizations.** Genomic DNA was extracted from strain zrk29 cells cultured in rich medium for ten days at 28 °C. The DNA library was prepared and sequenced using the Ligation Sequencing Kit (SQK-LSK109) and FLO-MIN106 vR9.4 flow-cell for 48 hours on MinKNOWN software v1.4.2 (Oxford Nanopore Technologies (ONT), United Kingdom), respectively. Then the whole-genome sequence determinations of strain zrk29 were carried out both with the Illumina MiSeq sequencing platform (San Diego, CA) and Oxford Nanopore MinION (Oxford, United Kingdom). A hybrid approach was utilized for genome assembly by using reads from both platforms and base-calling was performed using the Albacore software v2.1.10 (Oxford Nanopore Technologies). The nanopore reads were processed using the protocols toolkit for quality control and downstream analysis (1). And filtered reads were assembled by Canu version 1.8 (2), using the default parameters for Nanopore data. Finally, the genome was assembled into a single contig and manually circularized by deleting an overlapping end.

The genome relatedness values were calculated by multiple approaches: Amino acid identity (AAI), Average Nucleotide Identity (ANI) based on the MUMMER ultra-rapid aligning tool (ANIm), ANI based on the BLASTN algorithm (ANIb), the tetranucleotide signatures (Tetra), and *in silico* DNA–DNA similarity. The AAI values were calculated by AAI-profiler (http://ekhidna2.biocenter.helsinki.fi/AAI/) (3). ANIm, ANIb, and Tetra frequencies were calculated using the JSpecies WS (http://jspecies.ribohost.com/jspeciesws/) (4). The in silico DNA-DNA similarity values were calculated by the Genome-to-Genome Distance Calculator (GGDC) (<http://ggdc.dsmz.de/>) (5).

**Real-Time Quantitative Reverse Transcription PCR (qRT-PCR).**

For qRT-PCR, cells of strain zrk29 cultured in 1.5 L basal medium supplemented with or without 100 µg/ml *E. coli* genomic DNA and 100 µg/ml RNA for seven days was respectively collected at 8000 × *g* for 20 minutes. Total RNAs from each sample were extracted using the Trizol reagent (Solarbio, China) and the RNA concentration was measured using Qubit® RNA Assay Kit in Qubit® 2.0 Flurometer (Life Technologies, CA, USA). Then RNAs from corresponding sample were reverse transcribed into cDNA and the transcriptional levels of different genes were determined by qRT-PCR using SybrGreen Premix Low rox (MDbio, China) and the QuantStudioTM 6 Flex (Thermo Fisher Scientific, USA). The PCR condition was set as following: initial denaturation at 95 °C for three minutes, followed by 40 cycles of denaturation at 95 °C for 10 seconds, annealing at 60 °C for 30 seconds, and extension at 72 °C for 30 seconds. 16S rRNA gene was used as an internal reference and the gene expression was calculated using the 2^-ΔΔCt^ method, with each transcript signal normalized to that of 16S rRNA. Transcript signals for each treatment were compared to those of control group. Speciﬁc primers for genes within the locus of nucleic acid-degradation of strain zrk29 and 16S rRNA gene were designed using Primer 5.0 as shown in Supplementary Table S9.

**A detailed procedure for transcriptomic sequencing analysis of *H. nucleasis* zrk29 cultured under different conditions.**

**(1) Library preparation for strand-specific transcriptome sequencing.** A total amount of 3 μg RNA per sample was used as input material for the RNA sample preparations. Sequencing libraries were generated using NEBNext^®^ Ultra™ Directional RNA Library Prep Kit for Illumina^®^ (NEB, USA) following manufacturer’s recommendations and index codes were added to attribute sequences to each sample. rRNA is removed using a specialized kit that leaves the mRNA. Fragmentation was carried out using divalent cations under elevated temperature in NEBNext First Strand Synthesis Reaction Buffer (5×). First strand cDNA was synthesized using random hexamer primer and M-MuLV Reverse Transcriptase (RNaseH^-^). Second strand cDNA synthesis was subsequently performed using DNA Polymerase I and RNase H. In the reaction buffer, dNTPs with dTTP were replaced by dUTP. Remaining overhangs were converted into blunt ends via exonuclease/polymerase activities. After adenylation of 3’ ends of DNA fragments, NEBNext Adaptor with hairpin loop structure was ligated to prepare for hybridization. In order to select cDNA fragments of preferentially 150~200 bp in length, the library fragments were purified with AMPure XP system (Beckman Coulter, Beverly, USA). Then 3 μL USER Enzyme (NEB，USA) was used with size-selected, adaptor-ligated cDNA at 37 °C for 15 minutes followed by five minutes at 95 °C before PCR. Then PCR was performed with Phusion High-Fidelity DNA polymerase, Universal PCR primers and Index (X) Primer. At last, products were purified (AMPure XP system) and library quality was assessed on the Agilent Bioanalyzer 2100 system.

**(2) Clustering and sequencing.** The clustering of the index-coded samples was performed on a cBot Cluster Generation System using TruSeq PE Cluster Kit v3-cBot-HS (Illumia) according to the manufacturer’s instructions. After cluster generation, the library preparations were sequenced on an Illumina Hiseq platform and paired-end reads were generated.

**(3) Data analysis.** Raw data (raw reads) of fastq format were firstly processed through in-house perl scripts. In this step, clean data (clean reads) were obtained by removing reads containing adapter, reads containing ploy-N and low quality reads from raw data. At the same time, Q20, Q30 and GC content the clean data were calculated. All the downstream analyses were based on the clean data with high quality. Reference genome and gene model annotation files were downloaded from genome website directly. Both building index of reference genome and aligning clean reads to reference genome were used Bowtie2-2.2.3 (6). HTSeq v0.6.1 was used to count the reads numbers mapped to each gene. And then FPKM of each gene was calculated based on the length of the gene and reads count mapped to this gene. FPKM, expected number of Fragments Per Kilobase of transcript sequence per Millions base pairs sequenced, considers the effect of sequencing depth and gene length for the reads count at the same time, and is currently the most commonly used method for estimating gene expression levels (7).

**(4) Differential expression analysis.** Differential expression analysis of two conditions/groups (two biological replicates per condition) was performed using the DESeq R package (1.18.0) (8). DESeq provide statistical routines for determining differential expression in digital gene expression data using a model based on the negative binomial distribution. The resulting *P*-values were adjusted using the Benjamini and Hochberg’s approach for controlling the false discovery rate. Genes with an adjusted *P*-value < 0.05 found by DESeq were assigned as differentially expressed. (For DEGSeq without biological replicates) Prior to differential gene expression analysis, for each sequenced library, the read counts were adjusted by edgeR program package through one scaling normalized factor. Differential expression analysis of two conditions was performed using the DEGSeq R package (1.20.0) (9). The *P* values were adjusted using the Benjamini & Hochberg method. Corrected *P*-value of 0.005 and log_2_ (Fold change) of 1 were set as the threshold for significantly differential expression.

**(5) GO and KEGG enrichment analysis of differentially expressed genes.** Gene Ontology (GO) enrichment analysis of differentially expressed genes was implemented by the GOseq R package, in which gene length bias was corrected (10). GO terms with corrected *P* value less than 0.05 were considered significantly enriched by differential expressed genes. KEGG is a database resource for understanding high-level functions and utilities of the biological system, such as the cell, the organism and the ecosystem, from molecular-level information, especially large-scale molecular datasets generated by genome sequencing and other high-throughput experimental technologies (http://www.genome.jp/kegg/) (11). We used KOBAS software to test the statistical enrichment of differential expression genes in KEGG pathways.

**Genome sequencing of bacteriophages.** To sequence these bacteriophage genomes, the phage genomic DNA was extracted from different purified phage particles. Briefly, 1 μg/ml DNase I and RNase A were added to the concentrated phage solution for nucleic acid digestion overnight at 37 °C. The digestion treatment was inactivated at 80 °C for 15 minutes, followed by extraction with a Viral DNA Kit (Omega Bio-tek, USA) according to the manufacturer's instructions. Then, the genome sequencing was performed by Biozeron Biological Technology Co.Ltd (Shanghai, China). The detailed process of library construction, sequencing, genome assembly and annotation is described below.

**(1) Library construction and Illumina HiSeq sequencing.** Briefly, for Illumina pair-end sequencing of each phage, 1.0 μg genomic DNA was used for the sequencing library construction. Paired-end libraries with insert sizes of ~ 400 bp were prepared following the standard procedure. The purified genomic DNA was sheared into smaller fragments with a desired size by Covaris, and blunt ends were generated using the T4 DNA polymerase. And the desired fragments were purified through gel-electrophoresis, then enriched and amplified by PCR. The index tag was introduced into the adapter at the PCR stage and we performed a library quality test. Finally, the qualified Illumina pair-end library was used for Illumina NovaSeq 6000 sequencing (150 bp*2, Shanghai BIOZERON Co., Ltd).

**(2) Genome assembly.** The raw paired end reads were trimmed and quality controlled by the Trimmomatic (version 0.36, http://www.usadellab.org/cms/uploads/supplementary/Trimmomatic) (12) with parameters (SLIDINGWINDOW: 4:15, MINLEN: 75). And then clean data were obtained and used for further analysis. We have used the ABySS software (http://www.bcgsc.ca/platform/bioinfo/software/abyss) to perform genome assembly with multiple-Kmer parameters and got the optimal results. The GapCloser software (https://sourceforge.net/projects/soapdenovo2/files/GapCloser/) was subsequently applied to fill up the remaining local inner gaps and correct the single base polymorphism for the final assembly results.

**(3) Genome Annotation.** For bacteriophages, these obtained genome sequences were subsequently annotated by searching these predicted genes against non-redundant (NR in NCBI, 20180814), SwissProt (release-2021_03, http://uniprot.org) (13), KEGG (Release 94.0, http://www.genome.jp/kegg/) (14), COG (update-2020_03, http://www.ncbi.nlm.nih.gov/COG) (15) and CAZy (update-2021_09, http://www.cazy.org/) (16) databases.

**A detailed procedure for metabolomic analysis of *Hujiaoplasma nucleasis* zrk29 cultured under different pressures.**

**(1)** **Metabolites Extraction.** The samples were placed in the tubes and then resuspended with prechilled 80% methanol and 0.1% formic acid by well vortex. After that, the samples were melted on ice and whirled for 30 seconds, ultrasonic crushing for six minutes and centrifuged at 5000 × *g* for one minute. And then, the supernatant was freeze-dried and dissolved with 10% methanol. Finally, the solution was injected into the LC-MS/MS system analysis (17, 18).

**(2) UHPLC-MS/MS Analysis.** UHPLC-MS/MS analyses were performed using a Vanquish UHPLC system (ThermoFisher, Germany) coupled with an Orbitrap Q ExactiveTMHF-X mass spectrometer (Thermo Fisher，Germany) in Novogene Co., Ltd. (Beijing, China). Samples were injected onto a Hypesil Gold column (100×2.1 mm, 1.9 μm) using a 17 minutes linear gradient at a flow rate of 0.2 ml/min. The eluents for the positive polarity mode were eluent A (0.1% FA in Water) and eluent B (methanol).The eluents for the negative polarity mode were eluent A (5 mM ammonium acetate, pH=9.0) and eluent B (methanol). The solvent gradient was set as follows: 2% B, 1.5 min; 2-100% B, 12.0 min; 100% B, 14.0 min；100-2% B, 14.1 min；2% B, 17 min. Q Exactive TMHF-X mass spectrometer was operated in positive/negative polarity mode with spray voltage of 3.2 kV, capillary temperature of 320 °C, sheath gas flow rate of 40 arb and aux gas flow rate of 10 arb.

**(3) Data processing and metabolite identification.** The raw data files generated by UHPLC-MS/MS were processed using the Compound Discoverer 3.1 (CD3.1, ThermoFisher) to perform peak alignment, peak picking, and quantitation for each metabolite. The main parameters were set as follows: retention time tolerance, 0.2 min; actual mass tolerance, 5 ppm; signal intensity tolerance, 30%; signal/noise ratio, 3; and minimum intensity, 100,000. After that, peak intensities were normalized to the total spectral intensity. The normalized data was used to predict the molecular formula based on additive ions, molecular ion peaks and fragment ions. And then peaks were matched with the mzCloud (https://www.mzcloud.org/), mzVaultand Mass List database to obtain the accurate qualitative and relative quantitative results. Statistical analyses were performed using the statistical software R (version R-3.4.3), Python (version 2.7.6) and CentOS (release 6.6). When these data were not normally distributed, normal transformations were attempted using of area normalization method.

**(4) Data Analysis.** These metabolites were annotated using the KEGG database (https://www.genome.jp/kegg/pathway.html), HMDB database (https://hmdb.ca/metabolites) and LIPIDMaps database (http://www.lipidmaps.org/). Principal components analysis (PCA) and partial least squares discriminant analysis (PLS-DA) were performed at metaX (a flexible and comprehensive software for processing metabolomics data). We applied univariate analysis (t-test) to calculate the statistical significance (*P*-value).The metabolites with VIP > 1 and *P*-value< 0.05 and fold change≥2 or FC≤0.5 were considered to be differential metabolites.

**Measurement of biofilm formation of *Pseudomonas aeruginosa* PAO1 incubated in the medium supplemented with or without the supernatant of strain zrk29.** To check the anti-biofilm activity of the supernatant of strain zrk29 against *Pseudomonas aeruginosa* PAO1, as described previously (19), the overnight culture of PAO1 was diluted to OD_600_ of 0.1 with LB medium (10 g/l peptone, 5 g/l yeast extract, 10 g/l NaCl, pH adjusted to 7.0), and 200, 180, 150 µl of fresh diluent was incubated statically in a 96-well polystyrene plate respectively supplemented with 0, 20, 50 µl of the supernatant of strain zrk29 at 28 °C for 24 hours. In parallel, the medium without cells was used as the control group. To quantify the amount of biofilm formation, the planktonic bacterial cells were abandoned, and the wells were rinsed gently with sterile distilled water and subsequently stained with 1% crystal violet for 10 minutes. The stained biofilm was dissolved in 200 μl ethanol (100%, volume/volume) and detected by spectrophotometry at 595 nm (20). Each experiment was repeated three times.

**Statistical analysis.** Statistical analysis in this study was performed using GraphPad Prism 5 with one-way analysis of variance (one-way ANOVA) and multiple comparisons. A statistically signiﬁcance was deﬁned in this study by *P* < 0.05 (indicated by *), *P* < 0.01 (indicated by **) or *P* < 0.001 (indicated by ***).

**REFERENCES**

1. Loman NJ, Quinlan AR. 2014. Poretools: a toolkit for analyzing nanopore sequence data. *Bioinformatics* 30:3399-3401.

2. Koren S, Walenz BP, Berlin K, Miller JR, Bergman NH, Phillippy AM. 2017. Canu: scalable and accurate long-read assembly via adaptive k-mer weighting and repeat separation. *Genome Res* 27:722-736.

3. Medlar AJ, Toronen P, Holm L. 2018. AAI-profiler: fast proteome-wide exploratory analysis reveals taxonomic identity, misclassification and contamination. *Nucleic Acids Res* 46:W479-W485.

4. Richter M, Rossello-Mora R, Glockner FO, Peplies J. 2016. JSpeciesWS: a web server for prokaryotic species circumscription based on pairwise genome comparison. *Bioinformatics* 32:929-931.

5. Meier-Kolthoff JP, Auch AF, Klenk HP, Goker M. 2013. Genome sequence-based species delimitation with confidence intervals and improved distance functions. *BMC Bioinf* 14:60.

6. Langmead B, Salzberg SL. 2012. Fast gapped-read alignment with Bowtie 2. *Nat Methods* 9:357-U54.

7. Trapnell C, Pachter L, Salzberg SL. 2009. TopHat: discovering splice junctions with RNA-Seq. *Bioinformatics* 25:1105-1111.

8. Anders S, Huber W. 2010. Differential expression analysis for sequence count data. *Genome Biol* 11.

9. Wang LK, Feng ZX, Wang X, Wang XW, Zhang XG. 2010. DEGseq: an R package for identifying differentially expressed genes from RNA-seq data. *Bioinformatics* 26:136-138.

10. Young MD, Wakefield MJ, Smyth GK, Oshlack A. 2010. Gene ontology analysis for RNA-seq: accounting for selection bias. *Genome Biol* 11.

11. Kanehisa M, Araki M, Goto S, Hattori M, Hirakawa M, Itoh M, Katayama T, Kawashima S, Okuda S, Tokimatsu T, Yamanishi Y. 2008. KEGG for linking genomes to life and the environment. *Nucleic Acids Res* 36:D480-D484.

12. Bolger AM, Lohse M, Usadel B. 2014. Trimmomatic: a flexible trimmer for Illumina sequence data. *Bioinformatics* 30:2114-20.

13. Consortium U. 2021. UniProt: the universal protein knowledgebase in 2021. *Nucleic Acids Res* 49:480-489.

14. Kanehisa M, Furumichi M, Sato Y, Ishiguro-Watanabe M, Tanabe M. 2021. KEGG: integrating viruses and cellular organisms. *Nucleic Acids Res* 49:545-551.

15. Galperin MY, Wolf YI, Makarova KS, Vera Alvarez R, Landsman D, Koonin EV. 2021. COG database update: focus on microbial diversity, model organisms, and widespread pathogens. *Nucleic Acids Res* 49:274-281.

16. Drula E, Garron ML, Dogan S, Lombard V, Henrissat B, Terrapon N. 2022. The carbohydrate-active enzyme database: functions and literature. *Nucleic Acids Res* 50:571-577.

17. Sellick CA, Hansen R, Stephens GM, Goodacre R, Dickson AJ. 2011. Metabolite extraction from suspension-cultured mammalian cells for global metabolite profiling. *Nat Protoc* 6:1241-9.

18. Yuan M, Breitkopf SB, Yang X, Asara JM. 2012. A positive/negative ion-switching, targeted mass spectrometry-based metabolomics platform for bodily fluids, cells, and fresh and fixed tissue. *Nat Protoc* 7:872-81.

19. Wu S, Liu G, Zhang D, Li C, Sun C. 2015. Purification and biochemical characterization of an alkaline protease from marine bacteria *Pseudoalteromonas* sp. 129-1. *J Basic Microbiol* 55:1427-34.

20. Lee KJ, Kim JA, Hwang W, Park SJ, Lee KH. 2013. Role of capsular polysaccharide (CPS) in biofilm formation and regulation of CPS production by quorum-sensing in *Vibrio vulnificus*. *Mol Microbiol* 90:841-57.
