# Supplementary material for: Mechanisms of nucleic acid degradation and high hydrostatic pressure tolerance of a novel deep-sea wall-less bacterium
Source: mBio. 2023 Aug 8;14(4):e00958-23. doi: 10.1128/mbio.00958-23 (PMC10470597; doi:10.1128/mbio.00958-23)
Supplement: Supplemental Tables — Tables S1-S9. [file mbio.00958-23-s0004.docx]

**Table S1.** Genomic features of *H. nucleasis* zrk29 with *Izemoplasmataceae* strains zrk13, HR1, HR2, and ZiA1.

| Feature | zrk29 | zrk13 | HR1 | HR2 | ZiA1 |
| --- | --- | --- | --- | --- | --- |
| Gene Bank ID | CP051151 | CP048914 | CP009415 | JRFF00000000 | NQYJ00000000 |
| Genome size (bp) | 1,808,583 | 1,958,905 | 1,878,735 | 2,115,618 | 1,884,011 |
| G+C content (%) | 30.7 | 38.2 | 31.3 | 29.2 | 29.6 |
| No.scaffolds/contigs | 1 | 1 | 1 | 78 | 22 |
| No. of genes | 1,691 | 1,872 | 1,846 | 2,284 | 1,877 |
| No. of rRNAs | 6 | 3 | 4 | 3 | 0 |
| No. of tRNAs | 37 | 42 | 38 | 58 | 34 |
| No. of protein-coding genes | 1,568 | 1,762 | 1,794 | 2,222 | 1,803 |
| Completeness (%) | 100 | 100 | 100 | 92.1 | 98.7 |

**Table S2.** The amino acid identity (AAI) values among strains zrk29, zrk13, HR1, HR2, and ZiA1.

| AAI (%) | zrk29 | zrk13 | HR1 | HR2 | ZiA1 |
| --- | --- | --- | --- | --- | --- |
| zrk29  zrk13  HR1 | 100  53.4  58.4 | 53.4  100  69.4 | 58.4  69.4  100 | 58.3  67.0  69.0 | 58.8  64.6  66.0 |
| HR2 | 58.3 | 67.0 | 69.0 | 100 | 66.8 |
| ZiA1 | 58.8 | 64.6 | 66.0 | 66.8 | 100 |

**Table S3.** The average nucleotide identities (ANIb) among strains zrk29, zrk13, HR1, HR2, and ZiA1.

| ANIb (%) | zrk29 | zrk13 | HR1 | HR2 | ZiA1 |
| --- | --- | --- | --- | --- | --- |
| zrk29 | 100 | 64.61 | 65.58 | 65.78 | 65.59 |
| zrk13 | 64.11 | 100 | 68.59 | 67.18 | 66.40 |
| HR1 | 65.25 | 68.72 | 100 | 70.75 | 69.16 |
| HR2 | 65.62 | 67.42 | 70.83 | 100 | 69.86 |
| ZiA1 | 65.66 | 66.57 | 69.43 | 70.17 | 100 |

**Table S4.** The average nucleotide identities (ANIm) among strains zrk29, zrk13, HR1, HR2, and ZiA1.

| ANIm (%) | zrk29 | zrk13 | HR1 | HR2 | ZiA1 |
| --- | --- | --- | --- | --- | --- |
| zrk29 | 100 | 83.86 | 83.87 | 82.84 | 82.66 |
| zrk13 | 83.86 | 100 | 85.94 | 86.53 | 81.98 |
| HR1 | 83.87 | 85.94 | 100 | 82.43 | 81.57 |
| HR2 | 82.87 | 86.53 | 82.47 | 100 | 100 |
| ZiA1 | 82.66 | 81.98 | 81.55 | 81.76 | 81.76 |

**Table S5.** The Tetra values among strains zrk29, zrk13, HR1, HR2, and ZiA1.

| Tetra | zrk29 | zrk13 | HR1 | HR2 | ZiA1 |
| --- | --- | --- | --- | --- | --- |
| zrk29 | 1 | 0.82018 | 0.81311 | 0.80192 | 0.82059 |
| zrk13 | 0.82018 | 1 | 0.85192 | 0.77694 | 0.837 |
| HR1 | 0.81311 | 0.85192 | 1 | 0.92457 | 0.94627 |
| HR2 | 0.80192 | 0.77694 | 0.92457 | 1 | 0.92438 |
| ZiA1 | 0.82059 | 0.837 | 0.94627 | 0.92438 | 1 |

**Table S6.** The *in silico* DDH estimates among strains zrk29, zrk13, HR1, HR2, and ZiA1.

| GGDC (%) | zrk29 | zrk13 | HR1 | HR2 | ZiA1 |
| --- | --- | --- | --- | --- | --- |
| zrk29 | 100 | 26.90 | 18.90 | 25.30 | 17.20 |
| zrk13 | 26.90 | 100 | 17.50 | 18.50 | 15.80 |
| HR1 | 18.90 | 17.50 | 100 | 17.50 | 16.00 |
| HR2 | 25.30 | 18.50 | 17.50 | 100 | 16.70 |
| ZiA1 | 17.20 | 15.80 | 16.00 | 16.70 | 100 |

**Table S7.** Physiological characteristics of *H. nucleasis* zrk29 and *Izemoplasmataceae* strain zrk13.

| **Characteristic** | **zrk29** | **zrk13** |
| --- | --- | --- |
| Cell morphology  Cell length (µm)  Optimum temperature (°C)  Optimum pH  Optimum NaCl concentration (%)  Utilization as a sole carbon source:  Glucose  Maltose  Butyrate  Fructose  Sucrose  Acetate  Formate  Starch  Isomaltose  Trehalose  Galactose  Cellulose  Xylose  Lactate  Ethanol  D-mannose  Glycerin  Rhamnose  Sorbitol  DNA G+C content (mol%)  Isolation source | coccoid  0.3-0.5  28-32  7.0  0-4  +  +  -  +  +  +  +  -  +  -  -  -  -  +  -  +  +  +  -  30.68%  deep-sea  sediments | coccoid  0.3-0.8  28  7.0  0-4  +  +  +  +  +  +  +  +  +  +  -  -  -  +  +  +  +  +  -  38.21%  deep-sea sediments |

**Table S8.** Primers used for qRT-PCR for detecting the growth of *H. nucleasis* zrk29.

| **Primer name** | **Nucleotide Sequence (5’-3’)** |
| --- | --- |
| zrk29-f | TGTAGCGGTGGAATGCGTAG |
| zrk29-r | TTTACGGCGTGGACTACCAG |

**Table S9.** Primers used for qRT-PCR for detecting the expression of genes within the locus of nucleic acid-degradation in *H. nucleasis* zrk29.

| **Primer name** | **Nucleotide Sequence (5’-3’)** |
| --- | --- |
| 1-f | TCGCTATCATCTTCATCTTCGTCT |
| 1-r | AGACAAGCCATTGATCTTTGGGA |
| 2-f | CAAGGTTTTTAAGGCCCCGC |
| 2-r | CCCGATTATCTTTGTGGGTGC |
| 3-f | TCCGCATCAGCCAATAACTCA |
| 3-r | CGTTCCAAAGGGCCAGCTAT |
| 4-f | TCCAGCCATCTGTCCTATCAAAC |
| 4-r | GAAGCATGTGGCCTATCGGA |
| 5-f | AGATCCAACAACCGAACTTCCA |
| 5-r | TTGGAGCAACTGTGGGTTCT |
| 6-f | AGTGTTCCAGCATCAGCTTCA |
| 6-r | TACGGCGCAAGCTTAGTTGA |
| 7-f | CGGCCAAAGGCATCTTCAAC |
| 7-r | AAAGCATATGCAGCGCAAGC |
| 8-f | CCTCTACAGTTTCAAAAGCCGC |
| 8-r | GCAGTCATCAATACAGCGCC |
| 9-f | TTTCGTACCGCTTCGTCCAA |
| 9-r | TCAAGAAGGAACTGGACGTGT |
| 10-f | ACACGTTTCTTGCTGACTTGC |
| 10-r | ATCGGCGCTAACGGTAACTT |
| 11-f | CGCATTTGCAATCTTAAACGAGC |
| 11-r | TCAGGTGGACAGCAACAACG |
| 12-f | GCCCCAAAAACAATGCCGAA |
| 12-r | ATGAGAATTTGCCTGAAGATGAGTT |
| 13-f | TCGGGCAAAACAACAGAAGC |
| 13-r | AGAAGCTCCAACTCAAGAACCA |
| 14-f | AAAATGCACCAGCTAGCACTG |
| 14-r | GGGCTGCCAGTCTGGTTTTA |
| 15-f | TTCAATCCGACAGGTACACCA |
| 15-r | TCGACTCAAGAAGGCGAAACA |
| 16-f | CCAGGGATAGACATGCCTTGA |
| 16-r | GGAGATATGGTGGTAGCCGC |
| 17-f | TTTCACAAACGTTACACGGGTT |
| 17-r | ATTCAGCGGACCTAGAGGGA |
